# Cardioprotection Through Mitochondrial Modulation: A Systematic Review of Pharmacological Interventions in Animal Models of I/R Injury

**DOI:** 10.1007/s10557-026-07851-0

**Published:** 2026-03-04

**Authors:** Trissha Ybanez, Joshua Ingles, Eugene Du Toit, Jason Peart

**Affiliations:** 1 School of Pharmacy and Medical Science, Griffith University, Southport, QLD 4217 Australia; 2 Institute for Biomedicine and Glycomics, Griffith University, Southport, QLD 4215 Australia

**Keywords:** Cardiac ischaemia, Mitochondria, Mitochondrial dysfunction, Cardioprotection

## Abstract

**Purpose:**

Myocardial reperfusion following ischaemia paradoxically exacerbates mitochondrial and thus cardiac dysfunction. Although various treatment strategies have been utilised to prevent irreversible myocardial injury, translation to positive clinical trial outcomes has been unsuccessful. This systematic review aimed to evaluate pharmacological interventions in animal models of ischaemia–reperfusion (I/R), with emphasis on cardiac and cell death outcomes and direct assessment of mitochondrial bioenergetics.

**Methods:**

Search terms were entered into PubMed, Scopus, Embase and Web of Science. Screening, data extraction and quality assessment of papers were conducted according to the inclusion and exclusion criteria selected for this study. Eighteen papers from a total of 1571 studies were included. These studies investigated 15 drugs of interest in animals subjected to either in vivo or ex vivo I/R. Mitochondrial function parameters were assessed by measuring either mitochondrial respiration and/or enzyme activity, with 4 of these also assessing electron transport chain protein expression.

**Results:**

Pharmaceuticals preserved mitochondrial respiratory capacity by directly targeting the electron transport chain complexes or indirectly via proteins involved in canonical cardioprotective pathways. This led to improved post-ischaemic cardiac function and reductions in markers of cellular injury and myocardial infarction.

**Conclusion:**

Multi-targeted manipulation of components of mitochondrial signalling and function evidently reduces I/R injury. Quality assessment of most papers revealed an unclear risk of bias due to inadequate reporting of study parameters. Clear and consistent reporting of study outcomes, specifically mitochondrial bioenergetics across all experimental stages, is essential to enhance the translational potential of mitoprotective compounds in the clinical treatment of I/R.

**Supplementary Information:**

The online version contains supplementary material available at https://doi.org/10.1007/s10557-026-07851-0.

## Introduction

Ischaemic heart disease (IHD) continues to be the leading cause of death worldwide. The Global Burden of Disease, Injuries, and Risk Factors (GBD) study reported an average of 9.4 million deaths caused by IHD in the year 2021 [1]. Recently, the World Heart Federation 2023 report also identified IHD as the leading cause of premature death in men in 146 countries, and in women in 98 countries [2]. Acute myocardial infarction (AMI), also known as a heart attack, is the most lethal consequence of IHD and requires the immediate restoration of blood flow to prevent irreversible myocardial damage [3]. Hence, new strategies are needed to protect the myocardium during acute ischaemia and following revascularisation, to combat the ever-increasing global health burden.

An ischaemic heart is characterised by reduced blood supply to the myocardium, usually originating from, but not limited to, the formation of an atherosclerotic plaque in the coronary arteries [4]. Over time, the plaque grows exponentially, and as inflammation worsens, the fibrous cap ruptures, facilitating thrombus formation and impeding blood flow to the myocardium [5]. The nutrient and oxygen deficiency shuts down oxygen-dependent adenosine triphosphate (ATP) production *via* mitochondrial oxidative phosphorylation. To meet the metabolic demands of the ischaemic tissue, anaerobic glycolysis dominates. However, this process leads to excessive lactate production and the accumulation of reactive oxygen species (ROS), as well as phosphate, sodium, and calcium ions, which compromise cell membrane integrity and result in cellular necrosis without timely reperfusion [6]. Prolonged ischaemia causes irreversible tissue damage and ultimately leads to AMI. While reintroducing blood flow to the myocardium after an ischaemic episode remains the gold standard for preventing permanent tissue damage, it paradoxically induces an additional form of myocardial injury known as ischaemia-reperfusion (I/R) injury.

Timely reperfusion resolves oxygen deprivation but does not compensate for mitochondrial damage sustained during prolonged ischaemia [7]. Thus, the compromised system cannot efficiently cope with the increased influx of substrates upon reperfusion. Rapid re-energisation of the mitochondria abruptly increases ATP production and heightens proton leak and the generation of free radicals from the mitochondrial electron transport chain (ETC) complexes [8]. The mitochondria ultimately become dysfunctional, triggering the opening of the mitochondrial permeability transition pore (mPTP) [9]. This leads to cytochrome c release, activation of pro-apoptotic caspases, and promotes the loss of mitochondrial membrane potential – a process thought to be the primary gateway towards irreversible cell death and infarction [10].

Various cardioprotective strategies have been implemented over the years to prevent I/R injury, such as mechanical manoeuvres involving brief, sublethal cycles of ischaemia and reperfusion, collectively referred to as ischaemic conditioning. It involves intermittent coronary artery ligation and release before (ischaemic preconditioning) [11] and after ischaemia (ischaemic post-conditioning) [12] and can also be done remotely *via* repeated inflation and deflation of a blood pressure cuff, known as remote ischaemic conditioning [13, 14]. Cardioprotection harnessed by ischaemic conditioning involves the activation of pro-survival signalling pathways that ultimately converge to protect mitochondria [15]. Years of research into ischaemic conditioning have yielded substantial mechanistic insights into cardioprotective pathways, leading to the development of pharmacological conditioning, which aims to target canonical cardioprotective pathways by inhibiting pro-death signalling or enhancing pro-survival pathways [16].

As the heart’s primary source of energy and a key mediator of cardioprotective signalling, the mitochondria are an ideal target for pharmacological manipulation [17]. However, translating numerous pharmacological compounds into the clinical setting remains a challenge despite promising results at the preclinical level [18, 19]. A greater translational gap exists in mitochondria-targeted therapies, as earlier preclinical studies primarily focused on cardiac function and other mitochondrial endpoints while overlooking direct investigation of mitochondrial bioenergetic outcomes, such as mitochondrial respiration and electron transport chain activity. This results in an incomplete understanding of the drugs under investigation and their impacts on mitochondrial function, which may contribute to apparent underwhelming clinical trial outcomes.

Accordingly, this systematic literature review aims to evaluate pharmacological interventions tested in animal models of I/R that employed direct measurements of mitochondrial bioenergetics as primary indicators of mitochondrial function. The scope was also intentionally restricted to mechanistic studies conducted in the absence of comorbid conditions. By synthesising preclinical studies that assessed mitochondrial bioenergetics alongside cardiac outcomes, this review enables a critical appraisal of pharmacological mechanisms of action, efficacy, and study quality, which are essential steps in advancing mitoprotective compounds for the clinical treatment of I/R.

## Methods

### Search Strategy

The method of reporting and the structure of this systematic review adhere to the Preferred Items for Systematic Reviews and Meta-Analyses (PRISMA) 2020 guidelines (SI 1) [20]. The PICOS method was used to develop the search strategy and study characteristics of this study:

- Population (P): Animals exposed to an I/R protocol in both in vivo and ex vivo conditions.
- Intervention (I): Animal studies involving the administration of pharmacological interventions protecting against I/R injury.
- Control (C): Animal studies in which the I/R group exposed to the pharmacological intervention is compared to the control I/R group with or without vehicle treatment.
- Outcomes (O): Animal studies assessing the cardiac functional response, extent of cell death, and mitochondrial electron transport chain function in I/R groups with or without the intervention.
- Study design (S): Animal studies assessing the mechanism of action of pharmacological treatments and their effects on the cardiac mitochondria of animals exposed to I/R.

A comprehensive listing of synonyms, spelling differences, MeSH, and Emtree terms were combined with Boolean terms and refined before database search. Composed search strings (SI 2) were trialled and translated using the Polyglot Search Translation automation tool [21]. Translated search strings were entered into their respective databases using the advanced search tool in PubMed, Scopus, Web of Science (Core Collection), and Embase (Elsevier) (last search: 14th of May 2024).

### Data Extraction

Title and abstract screening, full-text reading, data extraction, and risk-of-bias assessment were conducted by two independent reviewers (TY and JI). All results from the database search were downloaded and sorted into Covidence for preliminary abstract and title screening and full-text reading [22]. Details of selected papers were then entered into a Microsoft Excel template containing all study characteristics for data collation, extraction, and summary. Any inconsistencies during each screening and data extraction stage were resolved through discussion, consensus, or advice from a third person or an expert in the field (JP and EDT).

### Inclusion Criteria

Eligible studies included studies on animals exposed to an I/R protocol (in vivo or ex vivo) and drugs against I/R injury. The three primary outcomes investigated were the impacts of the drug on cardiac contractile function, mitochondrial function, and degree of cell death at the end of reperfusion in hearts exposed to I/R. Specifically, cardiac function end-point outcomes after I/R had at least two of the following cardiac haemodynamic parameters: (1) Left ventricular developed pressure (LVDP); (2) Rate pressure product (RPP); (3) Left Ventricular End Diastolic Pressure (LVEDP). Mitochondrial function can be assessed using various experimental parameters. In terms of primary mitochondrial outcomes, we acknowledge that there is no single consensus definition of ‘mitochondrial function’ in the literature. For the purposes of this review, mitochondrial function was defined as the capacity for the ETC to support oxidative phosphorylation and energy production during I/R. Hence, our study inclusion criteria for mitochondrial function were restricted to studies that directly measured mitochondrial ETC-related outcomes. These outcomes were descriptively discussed to inform the overall state of mitochondrial bioenergetics following pharmacological intervention:

1. Mitochondrial respiration: assessed as oxygen consumption, usually measured using respirometry assays in which the addition of ADP to substrates supporting mitochondrial activity stimulates oxygen consumption and ATP production, reflecting ETC-driven oxidative phosphorylation (see Table 1 for standard terminology).

**Table 1:** Standard terminologies in mitochondrial respirometry assays

Parameter	Definition	Functional Significance
State 2 respiration	Basal oxygen consumption before ADP	Reflects baseline electron transport and proton leak prior to ATP synthesis
State 3 respiration	Oxygen consumption after ADP addition	The capacity of mitochondria to produce ATP via oxidative phosphorylation, driven by complex I (CI-driven State 3 respiration) and/or complex II substrates (CII-driven State 3 respiration)
State 4 respiration	Measures oxygen consumption after ADP is exhausted	Reflects proton leak and mitochondrial uncoupling; elevated values indicate impaired efficiency
Respiratory control ratio (RCR)	Ratio of active to resting respiration **(**State 3/State 4)	An index of mitochondrial coupling and integrity; reduced RCR indicates mitochondrial dysfunction.
Respiratory control index (RCI)	Alternative term used by some studies to describe respiratory coupling (State 3/State 2)	Reflects efficiency of coupling between electron transport and ATP synthesis; reduced RCI indicates mitochondrial dysfunction.
ADP/O ratio	ATP formed per oxygen consumed	Measure of bioenergetic efficiency; reduced values indicate impaired efficiency of ATP production
2. Mitochondrial ETC enzymatic activity: measured using biochemical assays, as reported in the original studies, to quantify the activity of individual ETC complexes or respirasome activities.
3. Mitochondrial ETC protein expression: assessed by immunoblotting or related protein quantification techniques, usually used to support mitochondrial respiration and mitochondrial ETC enzymatic activity data.

In addition, included studies also investigated cardiac cell injury, including infarct size and relevant biomarkers of cell death including lactate dehydrogenase, creatine kinase, cardiac troponin, and apoptotic markers. Other cardiac and mitochondrial parameters were also noted but were only recorded and described as additional information to support and justify primary end-reperfusion outcomes.

### Exclusion Criteria

Studies that included subjects that were not animals, and investigated organs other than the heart, or were genetically modified (e.g., transgenic animal model, gain or loss-of-function animal models) were excluded. Papers primarily based on in vitro studies of I/R, as well as neonates and ageing models of I/R were excluded. Any chronic effects of the drug were not included. Hence, studies where drugs were administered chronically (e.g., days to months) or more than 24 h before the I/R protocol were excluded. Animal studies that included a dietary intervention or models with cardioprotective potential (i.e., hypothermia models) that may affect the drug’s independent actions and drug induced I/R models (i.e., isoproterenol) were also excluded. Studies that did not assess cardiac and mitochondrial function, and cellular injury after I/R, or did not have an I/R protocol, were excluded. Papers where outcomes reported were not due to the direct effects of the drug were also excluded. Investigating mitochondrial respiration in tissue and isolated mitochondria primarily involves respirometry, which assesses various parameters within a single run. To avoid inadequate analysis of the effects of drugs on post-I/R cardiac mitochondrial respiration, only studies with at least two parameters of mitochondrial oxygen consumption were included, and any studies investigating a single parameter (i.e., RCR or RCI alone) for this criterion were excluded. In addition, abstracts alluding to mitochondrial function that did not contain the parameters specified in the inclusion criteria were excluded after full-text screening. Any studies with in vitro components alongside animal studies were also accepted; however, only data from the animal studies were extracted. In addition, to uphold the ethical handling of animals and to eliminate the possible contribution of the methods of animal sacrifice in end outcomes, we excluded studies involving cervical dislocation, “a blow to the head/neck” and decapitation due to its effects on cardiac mitochondria involving changes to metabolites in the Kreb’s cycle [23] and nucleotide and purine degradation [24].

All studies retrieved by the search string were included, and no date limitations were applied. However, studies not published in English were excluded during title and abstract screening. To ensure the quality and accessibility of papers, studies that were not peer-reviewed (confirmed by Ulrich’s Web), retracted, had no DOI, no full text available or could not be obtained through the Griffith Library or any other legible sources were omitted. Literature reviews, systematic reviews, clinical trials, conference papers, and those without an abstract were also excluded.

### Risk of Bias

The methodological and reporting quality assessment of included papers was completed using the Systematic Review Centre for Laboratory Animal Experimentation (SYRCLE) risk of bias tool [25] and the Collaborative Approach to Meta-Analysis and Review of Animal Experimental Studies (CAMARADES) [26], which are risk of bias tools specific to animal studies. Both tools assessed selection, performance, detection, reporting, attrition bias, and other biases that affect study quality.

## Results

### Study Selection and Animal Characteristics

**Fig. 1:**
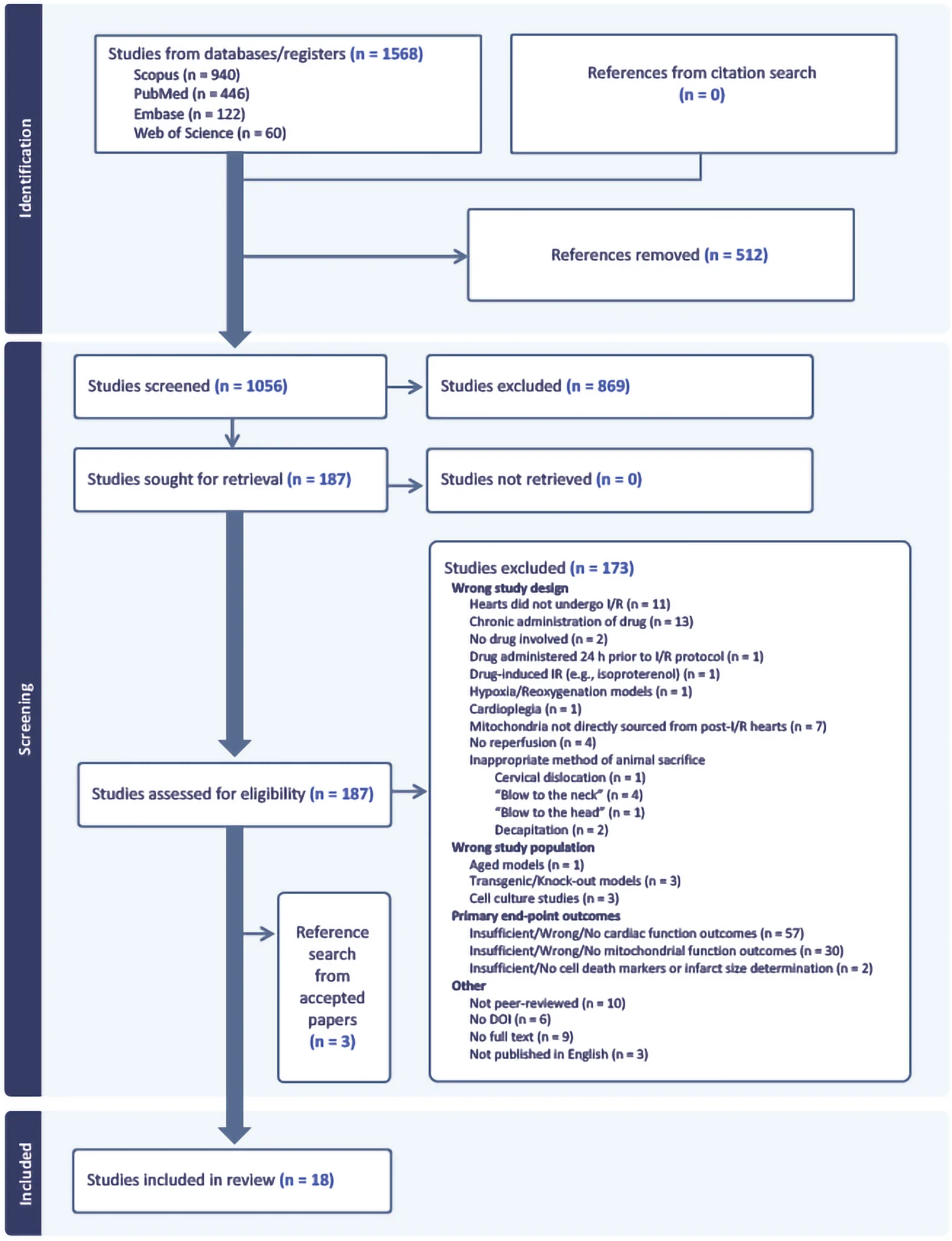
PRISMA flow diagram. Studies from Scopus, PubMed, Embase, and Web of Science were screened according to exclusion and inclusion criteria

Eighteen papers were included in the review from 1571 studies (1568 papers, including three studies from citation search in accepted papers), as detailed in the PRISMA flow diagram (Fig. 1). Interestingly, all included studies investigated male rodents only, with 94% of studies completed using rats, and a single study using C57BL/6J mice. Wistar rats were the most studied rat strain (53%), followed by Sprague-Dawley rats (41%), and one study was on Fischer-344 rats. Body weight was reported across 94% of the studies and ranged from 140 to 420 g for rats and 25 to 35 g for mice. Only 28% of studies reported age which ranged between 4 and 34 weeks old. Despite not filtering animal species, only rodent studies fit the inclusion criteria. Studies including other species and larger animals were identified but were rejected due to having insufficient mitochondrial and cardiac data. Animal characteristics are summarised in SI 3.

### Drug Characteristics and Ischaemia-Reperfusion Models

The primary drugs of interest and their optimal doses were extracted from each study (SI 4). These included sodium hydrogen sulfide (NaHS, 20 µM), 5-azacytidine (5 µM), sodium thiosulfate (STS, 1 mM, *n* = 4 studies), ARP-100 (10 µM), ONO-4817 (50 µM), amobarbital (2.5 mM), AVE-4890 (5 µM), angiotensin II (10 nM), metformin (200 mg/kg), diazoxide (100 µM), sappanone A (100 µM), cariporide (10 µM), metoprolol (10 µM), 3,4-dihydroxy-phenyl lactic acid (DLA, 5 mg/kg), and 5,5-dimethyl-1-pyrroline-*N*-oxide (DMPO, 1 mM). Sixteen studies used the Langendorff isolated heart model to assess the cardioprotective efficacy of the compounds via intracoronary perfusion. The remaining two studies utilised an in vivo model of I/R induced by left anterior descending coronary artery ligation with intravenous drug delivery. The ischaemic period ranged from 25 to 40 min, and the reperfusion period ranged from 30 to 120 min. Drug administration was performed either before prolonged ischaemia, or during ischaemia, or both.

Primary outcome measures were cardiac functional recovery and cellular injury (Table 2), and mitochondrial oxygen consumption, enzymatic activity and complex expression (Table 3). The summary of outcomes from the included studies focuses on comparisons of the individual effects of the primary drugs of interest alone versus the control I/R group. Co-treatment with another drug, or a more established I/R therapy (e.g., ischaemic preconditioning or ischaemic postconditioning), is occasionally mentioned when relevant to the interpretation of results. The most frequently recorded cardiac functional response was left ventricular developed pressure (LVDP) (89%, including those reported LVDP as a percentage of baseline function), followed by rate pressure product (61%, RPP), left ventricular end-diastolic pressure (56%, LVEDP), and rates of contraction and relaxation (50%, maximum and minimum dP/dt). Other parameters investigated from the ex vivo models were left ventricular end-systolic pressure (LVESP) and ischaemic contracture. Alongside these parameters, in vivo models also assessed arrhythmia scores, mean arterial pressure, stroke volume and ejection fraction after I/R. The most recorded mitochondrial outcome within the inclusion criteria was mitochondrial ETC activity (61% of studies), followed by mitochondrial respiration (56% of studies) and mitochondrial ETC protein expression (28% of studies). Other mitochondrial parameters, such as mitochondrial swelling, mitochondrial membrane potential, and other mitochondrial-related pathways and upstream pathways, were noted but were not considered primary outcomes. To aid interpretation of primary outcomes, results were synthesised based on shared pharmacological effects across cardiac, cellular, and mitochondrial outcomes rather than organised by individual studies. To support this, an integrated summary of mechanistic outcomes across all compounds is provided in Table 4. In terms of their classification according to their effect on mitochondrial function, compounds were categorised as either:

**Table 2:** Cardiac and cell death outcome following I/R

Drug Class	Compound	Cardiac functional outcomes	Cell death outcomes	References
Direct ETC Modulators	Amobarbital	↑ LVDP, RPP, ±dP/dT; ↓ LVEDP;	↓ IS, LDH	[27]
	Diazoxide	↑ LVDP, RPP; ↓ LVEDP; delayed ischaemic contracture	↓ IS	[28]
Sulfide Donors	NaHS	↑ LVDP, RPP; ↓ LVEDP	Pre-con:↓ IS; ↓ LDH, CK, DNA fragmentation Post-con: ↓ IS, LDH, DNA fragmentation	[29, 30]
	STS	Pre-con: ↑ LVDP, LSVP, RPP Post-con: ↑ LVDP (≈ in some studies), RPP; ≈ ±dP/dT and LVEDP	Pre-con: ↓ IS, LDH, CK (≈ in some studies) Post con: ↓ IS, LDH (↑ in some studies), CK, caspase 3 activity, DNA fragmentation	[31–34]
Ion Handling Modulators	Cariporide	↑ LVDP, ±dP/dT; ↓ LVEDP	↓ IS (ns when administered alone)	[36]
	Metoprolol	↑ LVDP, ±dP/dT; ↓ LVEDP	↓ IS	[36]
	AVE-4890	↑ LVDP, ↓ LVEDP	↓ LDH, cleaved PARP; ↑ AIF & EndoG in mitochondria; ↔ caspase-3	[35]
Canonical Pathway Modulators	Metformin	↑ LVDP (% baseline), RPP, LVESP, EF, SV; ↓ LVEDP, arrhythmia score; ↔ ±dP/dT	↓ IS; ↓ LDH; ↓ TUNEL; ↓ cleaved caspase-3/caspase-3; ↓ Bax; ↔ Bcl-2	[40, 41]
	5-Azacytidine	↑ LVDP, RPP, ±dP/dT	↓ LDH, CK, caspase-3 activity	[43]
	Angiotensin II	↑ LVDP, RPP, ±dP/dT; ↔ ischaemic contracture	↓ IS; ↓ LDH	[38]
	Sappanone A	↑ LVDP, ↑ ±dP/dT	↓ LDH, CK, cTnI	[42]
Antioxidants/Free Radical Scavengers	DLA	↓ LVEDP, LVESP, MAP, ±dP/dT	↓ IS; ↔ LDH; ↓ TUNEL; ↑ Bcl-2/Bax ratio; ↓ cleaved caspase-3	[37]
	DMPO	↑ LVDP (% baseline). RPP; ↓ LVEDP	↓ IS	[38]
MMP Inhibitors	ARP-100	↑ LVDP, ±dP/dT	↓ LDH	[39]
	ONO-4817			[39]

Outcomes refer to treatment vs. I/R group unless otherwise stated. Symbols: ± Minimum and maximum; ↑ Increased vs I/R group; ↓ Reduced vs I/R group; ≈ Comparable or near baseline (non-I/R) levels

**Table 3:** Mitochondrial outcomes following I/R

Drug class	Compound	Mitochondrial oxygen consumption (respirometry)	ETC enzymatic activity	ETC protein expression	References
Direct ETC modulator	Amobarbital	↑ CI-driven State 3, CI-driven RCR(in both SSM and IFM)	↑CI, ↑CI + III	—	[27]
	Diazoxide	↑ State 3; ↑ RCR; ↑ ADP-stimulated OXPHOS	↑ CII	—	[28]
Sulfide donors	NaHS	Pre-con: **≈** CI-driven State 3 respiration, CI-driven RCR, IFM ADP/O ion, CI-driven RCR, IFM ADP/O; ↓ SSM ADP/O Post-con: **≈** CI-driven RCR, ADP/O (in both subpopulations)	Pre-con: **≈** CI, CIII, CIV, CI+CIII, CII+CIII; ↔ CII Post-con: **≈** CI, CIII, CIV, CI+CIII, CII+CIII; ↓ CII*	—	[29, 30]
	STS	—	Pre-con: ↑ CI (SSM > IFM); ↔ CII; ↑ CIII, CIV (in both subpopulations); ↔ CII+CIII (in both subpopulation) Post-con: **≈** CI to CIV, CI+CIII, CII+CIII	Pre-con: ↓CI, CII, ↔ CV expression	[31–34]
Ion-handling modulators	Cariporide	↔ CI-driven State 3, CI-driven RCR	—	—	[36]
	Metoprolol	↑ CI-driven State 3, CI-driven RCR	—	—	[36]
	AVE-4890	↑ CII-driven State 3, CII-driven RCI	—	—	[35]
Canonical pathway modulators	Metformin	↑ CI-driven State 3, CI-driven RCI, CII-driven State 3, CII-driven RCI, CIV respiration, ADP/O	—	↔ CI to CIV, ↑ CV	[40, 41]
	5-Azacytidine	↑ CI-driven State 3, CI-driven RCR (in both SSM + IFM); ↔ CII-driven RCR, ↑ ADP/O (IFM only)	↑CI, CIV (both SSM and IFM)	—	[43]
	Angiotensin II	↑ CI-driven State 3, ↔ CI-driven RCI; synergistic ↑ when paired with IPC	—	—	[38]
	Sappanone A		↑CI to CIV	—	[42]
Antioxidant/Free radical scavengers	DLA	↑ State 3; ↑ RCR; improved OXPHOS efficiency	↑ CI	↑ CI (NDUFA10 subunit)	[37]
	DMPO	↑ CI-driven respiration; ↑ State 3; ↑ RCR	↑CI, CIV	↑CI, ↑CII, ↑CIV (CoXI, CoXV subunits)	[38]
MMP inhibitor	ARP-100	↑ CI-driven State 3, CI-driven RCR, CII-driven State 3, CII-driven RCR	—	—	[39]
	ONO-4817	↑ CI-driven State 3, CI-driven RCR, CII-driven State 3, CII-driven RCR	—	—	[39]

Outcomes refer to treatment vs. I/R group unless otherwise stated. Symbols: ± Minimum and maximum; ↑ Increased vs. I/R group; ↓ Reduced vs. I/R group; ≈ Comparable or near baseline (non-I/R) levels

- **Direct regulators of mitochondrial bioenergetics** (e.g. reversible complex I or complex II inhibition).
- **Indirect regulators of mitochondrial function**, which target mitochondrial dynamics, redox signalling, exhibit antioxidant and free radical scavenging activities, and preserve mitochondrial function by targeting upstream canonical pathways of cardioprotection.

### Effects of Pharmacological Interventions on Cardiac Function and Cell Death Parameters

#### Direct Modulators of Mitochondrial Bioenergetics

Amobarbital and diazoxide enhanced cardiac outcomes following ischaemia in Fischer-344 and Sprague-Dawley rats, respectively, when administered before ischaemia. Amobarbital, a reversible complex I inhibitor enhanced contractile function by a significant increase in LVDP, RPP and the heart’s rate of relaxation and contraction at the end of reperfusion when perfused 1 min before prolonged ischaemia [27]. This was accompanied by decreased diastolic pressure, infarct size and LDH levels at the end of reperfusion. Diazoxide (100 µM) reversibly inhibited complex II, delayed ischaemic contracture, and increased LVDP and RPP. This was associated with a decrease in LVEDP and a 73% reduction in infarct size at the end of reperfusion [28].

#### Indirect Modulators of Mitochondrial Function

##### Sulfide Donors

Sulfide-based drugs NaHS (20 μm) and STS (1 mM) consistently increased recovery of cardiac parameters while predominantly decreasing cell death markers in male Wistar rats. Both NaHS and STS administered before and after ischaemia resulted in significantly increased developed pressure, RPP, and in studies that measured it, systolic pressure, alongside reduced LVEDP at the end of reperfusion [29–34]. This was accompanied by a significant reduction in infarct size and lowered cell injury markers including lactate dehydrogenase (LDH) and creatine kinase (CK), while in studies that assessed DNA fragmentation, no damage was detected [29, 30, 34]. The timing of STS administration influenced cell death outcomes. For example, LDH levels were unaffected by STS postconditioning [34] but were reduced by STS preconditioning [31]. Overall, sulfide donors increased recovery of the heart following I/R and predominantly decreased necrotic cell death.

##### Ion Handling Modulators

Pre-ischaemic and post-ischaemic administration of AVE-4890, an NHE-1 inhibitor, was reported to also increase LVDP and decrease LVEDP at the end of reperfusion in isolated Sprague-Dawley rats hearts subjected to I/R [35]. It caused a significant reduction in LDH levels and in apoptotic markers such as cleaved poly-ADP ribose polymerase (PARP), but levels of caspase-3 remained unchanged compared to the I/R group in the cytosolic fraction. Despite this, other markers of apoptosis, such as apoptotic inducing factor and EndoG, remained in the mitochondria and had elevated protein expression levels in the organelle. Cariporide (10 µM) and metoprolol (10 µM), a sodium-hydrogen antiporter 1 (NHE-1) inhibitor and a beta-blocker, were used individually and in combination as a treatment against I/R injury in isolated Sprague-Dawley rat hearts [36]. Wang et al. found that I/R hearts exposed to either treatment had similar increases in LVDP and the rate of myocardial contraction and relaxation with a consequent decrease in diastolic pressure. Investigators also reported a reduction in infarct size for cariporide or metoprolol alone, but the effect was significant in I/R groups exposed to metoprolol only, but not cariporide alone. Collectively, treatments that prevented ion imbalance reduced cardiac dysfunction and prevented irreversible tissue damage.

##### Antioxidant/Free Radical Scavengers

Treatment with DLA at a 5 mg/kg dose in an in vivo I/R study using Sprague-Dawley rats resulted in attenuated infarct size and apoptosis markers [37]. However, the levels of coronary LDH release remained unchanged and comparable to those of the I/R group. Nevertheless, anti-apoptotic protein expression of B-cell lymphoma 2 (Bcl-2) was increased while pro-apoptotic Bax protein levels were diminished. This corresponded with the restoration of the Bcl-2/Bax ratio by DLA treatment. Additionally, DLA increased left-ventricular maximum and minimum developed pressures, leading to a reduction in end-diastolic pressure. Other cardiac functional parameters, such as mean arterial pressure and systolic pressure, were also restored by DLA treatment. In a study in which the free-radical scavenger spin trap 5,5-dimethyl-1-pyrroline N-oxide (DMPO), was administered at a concentration of 1 mM before ischaemia and during reperfusion to test its cardioprotective potential, it increased LVDP and RPP and significantly lowered LVEDP and infarct size in isolated Sprague-Dawley rat hearts [38].

##### Matrix Metalloproteinase Inhibitors

Matrix metalloproteinase-2 (MMPs) are endopeptidases activated by oxidative stress during I/R through proteolytic cleavage, resulting in NTT-MMP-2, a truncated isoform of MMP. Bassiouni et al. hypothesised that the NTT-MMP-2 isoform proteolyzes Mfn-2 during I/R [39]. Inhibitors specific to MMP, such as ARP-100 and ONO-4817, were used to test this hypothesis in male C57Bl/6J mice and showed significant improvements in LVDP and rates of left ventricular contraction and relaxation. A concomitant attenuation of LDH levels was also seen at the end of reperfusion.

##### Canonical Pathway and Metabolic Status Modulators

Metformin preserved cardiac functional recovery and reduced cell death in ex vivo and in vivo models of I/R. In isolated Sprague-Dawley rat hearts, metformin (2 mM) was perfused before ischaemia and throughout reperfusion, resulting in an overall increase in LVDP and RPP at the end of reperfusion [40]. In contrast, Palee et al. administered metformin (200 mg/kg) intravenously via the femoral vein in Wistar rats 15 min before coronary artery ligation. Echocardiography demonstrated significant improvements in RPP, LVESP, ejection fraction, and stroke volume, accompanied by reductions in LVEDP and arrhythmia score [41]. However, metformin did not affect the rates of contraction and relaxation. Despite differences in experimental model, route, and timing of administration, metformin lowered LDH levels, infarct size, and apoptotic markers, including caspase-3 and TUNEL-positive cells. Other apoptotic markers, such as Bcl-2 protein expression, remained unchanged, whereas Bax was significantly increased in the metformin groups compared with the control I/R group.

Similarly, pre-ischaemic perfusion with 5-azacytidine (5 µM) in isolated Wistar rat hearts increased LVDP and RPP, including the rate of contraction and relaxation of the I/R heart at the end of reperfusion. It also reduced infarct size, significantly attenuated LDH and CK levels, and lowered caspase-3 activity. Angiotensin II also increased post-ischaemic LVDP, RPP and rate of contraction, while ischaemic contracture was unaffected. Additionally, angiotensin II decreased both infarct size and LDH significantly [38]. On the other hand, sappanone A (100 µM) post-conditioning increased LVDP and the heart’s rates of contraction and relaxation, and significantly reduced cell death markers such as LDH, CK, and cardiac troponin I (cTnI) at the end of reperfusion [42].

### Effects of Pharmacological Interventions on Mitochondrial Bioenergetics

#### Mitochondrial Oxygen Consumption

Several compounds enhanced complex I-driven mitochondrial oxygen consumption, including NaHS, amobarbital metformin, 5-azacytidine, ARP-100, ONO-4817, and metoprolol. This was shown in increased State 3 respiration, respiratory control ratios (RCR) and adenosine diphosphate/oxygen (ADP/O) ratios [27, 29, 30, 36, 39–41, 43]. In studies using NaHS, complex I function was restored to near baseline levels, but did not yield a statistical difference when compared with I/R. This effect was more prominent in IFM than SSM. In contrast, treatment with 5-azacytidine significantly augmented complex I-driven respiration in both the IFM and SSM. Amobarbital treatment transiently inhibited complex I during ischaemia and preserved mitochondrial oxidative phosphorylation following I/R, evidenced by significantly higher complex I–linked State 3 respiration and RCR compared with the I/R group in both SSM and IFM [27]. These values were significantly higher than the I/R group but remained below the non-ischaemic time control group. Metalloproteinase inhibitors ARP-100 and ONO-4817 also increased complex-I driven state 3 respiration and RCR. In contrast, angiotensin II caused a non-significant increase in complex-I-driven respiration, with no impact on the RCI compared with the I/R group when administered alone [44].

Fewer compounds investigated respiration via complex II and complex IV. Complex II-driven RCR in both mitochondrial subpopulations remained unchanged with 5-azacytidine, with only the ADP/O ratio of the IFM responding favourably to the drug [43]. In contrast, AVE-4890, ARP-100, and ONO-4817 significantly increased complex II-driven State 3 respiration and associated RCR and RCI. Metformin also enhanced complex IV respiration accompanied by increases RCI and ADP/O ratios [40, 41]. Among the compounds, only cariporide did not affect mitochondrial oxygen consumption. Though, it increases complex I-linked respiration when combined with metoprolol.

#### Mitochondrial ETC Complex Activity

Across the included studies, several compounds improved activities of ETC complexes I–IV. Complex I activity was the most consistently enhanced target. NaHS restored complex I activity in both mitochondrial populations to near baseline levels, with respirasome activities (I + III and II + III) restored primarily in the IFM [29, 30]. STS likewise increased complex I activity, although the mitochondrial compartment affected differed by timing: postconditioning increased complex I activity [34], whereas pre-ischaemic STS improved complex I activity predominantly in the SSM [31]. Additional studies using STS reported broader improvements in enzyme activities across complexes I–IV under both pre- and postconditioning conditions [32, 33].

Other compounds also demonstrated improvements in complex I-linked activity. Treatment with 5-Azacytidine increased the activities of complex I and IV in both IFM and SSM [43]. On the other hand, amobarbital transiently inhibited complex I during ischaemia and preserved complex I activity and corresponding I + III respirasome activity in both mitochondrial subpopulations [27]. DLA restored complex I activity by enhancing NDUFA10 expression [37], while DMPO also increased the activities of complexes I and IV [38], and sappanone A increased complex I–IV activities [42]. Complex II activity was less consistently modulated. Treatment with NaHS did not restore complex II activity in either mitochondrial subpopulation [26] while STS decreased complex II activity despite improving complexes I, III, and IV [31, 34]. Diazoxide reversibly inhibited complex II during ischaemia, with activity returning upon reperfusion [28].

#### Mitochondrial ETC Protein Expression

Fewer studies assessed protein expression of ETC complexes. Administration of STS produced mixed effects: preconditioning decreased complex I and II protein levels, while complex V expression remained unchanged [31]. DLA increased complex I subunit NDUFA10 expression alongside enhanced complex I activity [37]. DMPO increased protein expression of complexes I, II, and IV [38] while metformin only enhanced complex V protein expression but not complex I to complex IV.

#### Influence of Upstream Pathways and Other Mitochondrial Parameters

Most studies modulated mitochondrial function indirectly through upstream cardioprotective signalling. The cardioprotective effects of STS preconditioning were attenuated when phosphoinositide 3-kinase (PI3K), mammalian target of rapamycin (mTOR), and mitochondrial ATP-sensitive potassium (mito-K_ATP_) channels were inhibited, suggesting a dependence on these pathways [33]. The molecular mechanisms underlying STS-mediated cardioprotection may differ between preconditioning or postconditioning protocols and require further investigation [33]. Similarly, 5-azacytidine involves PI3K, glycogen synthase kinase 3β (GSK3β), and the mito-K_ATP_ pathway [43], while the cardioprotective effects of DLA were abolished by sirtuin 1 (SIRT1) inhibition using sirtinol or EX-527 [37]. Metformin’s cardioprotective effect also differed between ex vivo and in vivo models. The effects of metformin in the ex vivo model were abolished by PPARα inhibition [40], while the in vivo model was dependent on AMPK, connexin-43 phosphorylation, PGC-1α upregulation and reduced Drp1 translocation [41]. On the other hand, angiotensin II improved cardiac recovery when administered alone by binding to AT-1 receptors but did not improve mitochondrial function [44]. However, when paired with IPC, angiotensin II improved complex I respiration by activating PKC.

Several studies also assessed additional mitochondrial parameters relevant to I/R injury, including outer membrane integrity, mitochondrial dynamics, mitochondrial biogenesis, mitochondrial calcium content and resistance to mPTP opening during I/R. Sappanone A, prevented mPTP opening, reduced mitochondrial ROS, promoted a shift toward mitochondrial fusion and modulated mitophagy markers, including reductions in PINK1 and p62 and increases in Parkin and LC3II in an AMPK-dependent [42]. Administration of STS before ischaemia increased mitochondrial copy number and mRNA levels of genes involved in ATP synthesis, consistent with increased PGC1α expression. On the other hand, MMP inhibitors increased ATP production during respiration (measured via fluorometric assay of respiration buffer after respirometry) and preserved mitochondrial mitofusin-2 (Mfn-2) protein levels [39]. Amobarbital preserved cytochrome c content, a marker of outer membrane integrity, in both the SSM and IFM [27]. In contrast, the cardioprotective effects of diazoxide were abolished by mito-K_ATP_ channel inhibitor 5-hydroxydecanoic acid (5-HD) [28]. Neither β-blockade with metoprolol nor NHE-1 inhibition with cariporide significantly reduced mitochondrial calcium accumulation following I/R. Overall, pharmacological agents target diverse upstream and other mitochondrial-related pathways, such as PI3K/Akt, mTOR, AMPK, mito-K_ATP_ channels, PGC1α, PPARα, SIRT1, and AT1R signalling, calcium handling and the mPTP (Table 4).

**Table 4:** Mechanistic classification of pharmacological drugs targeting mitochondrial function during I/R

Mechanistic Class	Compounds	Time of Administration	Primary Mechanistic Effect
Transient Electron Transport Chain Inhibitors	Amobarbital (CI inhibitor), Diazoxide (CII inhibitor)	Pre-ischaemia	Reversible inhibition of proximal ETC complexes reduces electron flux and ROS generation during ischaemia and preserves mitochondrial ETC integrity for reperfusion
Restoration of ETC activity	NaHS, STS, 5-Azacytidine, Sappanone A, DMPO, DLA	Pre-ischaemia or reperfusion	Restore or enhance ETC complex respiration and activity with antioxidant properties
Upstream Signalling Modulators	STS, 5-Azacytidine, Metformin, Sappanone A, AVE-4890	Pre-ischaemia or reperfusion	Activation of PI3K-Akt, AMPK-PGC1α-Sirt1 axis, GSK3β inhibition, mTOR signalling; regulate MQC; collectively results in ↑ mPTP threshold and mitochondrial function and integrity despite indirect ETC effects.
Ion-Handling Modulators	Cariporide, Metoprolol, AVE-4890	Pre-ischaemia	Reduces mitochondrial Ca²⁺ overload and mPTP opening
Adjunctive Synergy Mechanisms	Angiotensin II (paired with IPC)	Pre-ischaemia	PKC-dependent signalling increases CI-linked respiration only when combined with IPC

## Quality Assessment

**Fig. 2:**
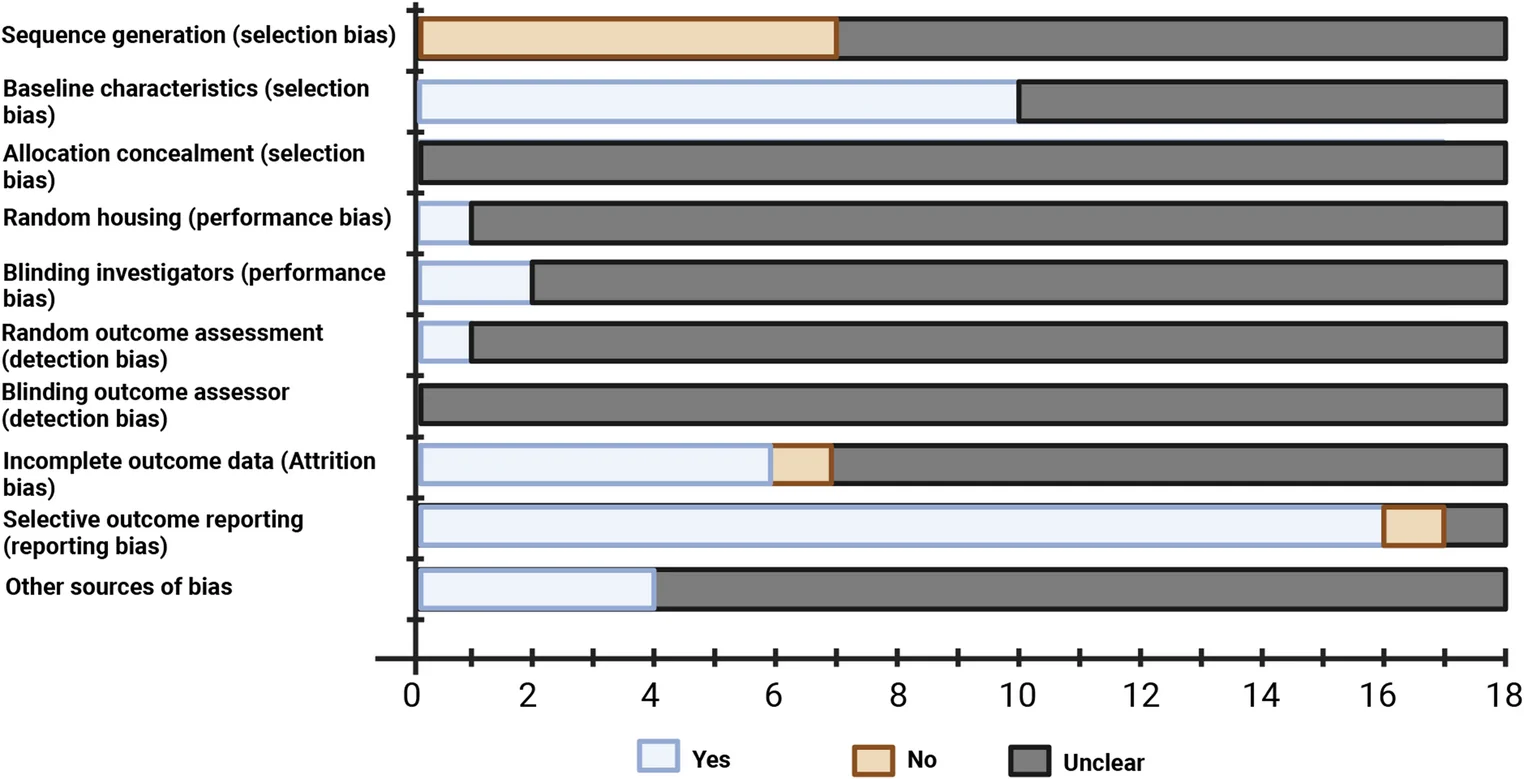
SYRCLE's risk of bias outcomes. Overview of quality assessment of 18 papers using the SYRCLE’s risk of bias tool. Papers were marked according to a set of questions specific to animal studies. Created in BioRender. Ybanez, T. (2026) https://BioRender.com/rq8r9pv

Most of the items in the SYRCLE’s risk of bias assessment tool were reported as “unclear” outcomes, resulting in an unknown risk of bias in most papers (Fig. 2). Selection bias was apparent in studies that did not mention or provide sufficient detail for sequence generation, baseline characteristics, and allocation concealment. In 39% of the studies, randomisation was not specified, while 61% cited a form of random allocation. However, the exact methods of randomisation were not disclosed. We considered pre-ischaemic cardiac functional parameters as baseline characteristics (heart rate, LVDP, minimum and maximum dP/dT, RPP, etc.). We found that 56% of the studies had similar baseline parameters, while 44% did not. Furthermore, none of the studies specified their group allocation methods and how different groups were concealed. Methods for animal housing, randomisation of investigator, and data analysis blinding were also unclear in all studies apart from one study that generated a randomised assignment list for post-perfusion outcome assessment. These observations suggest that most of these studies have an unknown risk for performance and detection bias. One study had incomplete outcome data without detailing the reason for this omission and is thus at a high risk of attrition bias. In contrast, 12 out of the 18 studies were reported as “unclear” due to inconsistent reporting or the absence of ‘n’ numbers for each data group in their figure or results section. Most studies (16 out of 18) recorded primary and secondary outcomes, with two studies at risk of selective outcomes reporting. In addition, one study had an inadequately detailed Langendorff isolated heart perfusion protocol, resulting in an unclear risk of reporting bias. We considered other sources of bias, such as possible pooling of drugs and inappropriate influence of study funders. We found that all studies adhered to the administration of a primary drug with no influence of other drugs. Studies involving pharmacological inhibition of multiple proteins to confirm their role in the cardioprotective pathway were conducted exclusively within designated inhibition groups. These compounds were neither classified as drug pooling nor considered the primary drugs of interest. Funding was also recorded and suitable for all studies.

**Fig. 3:**
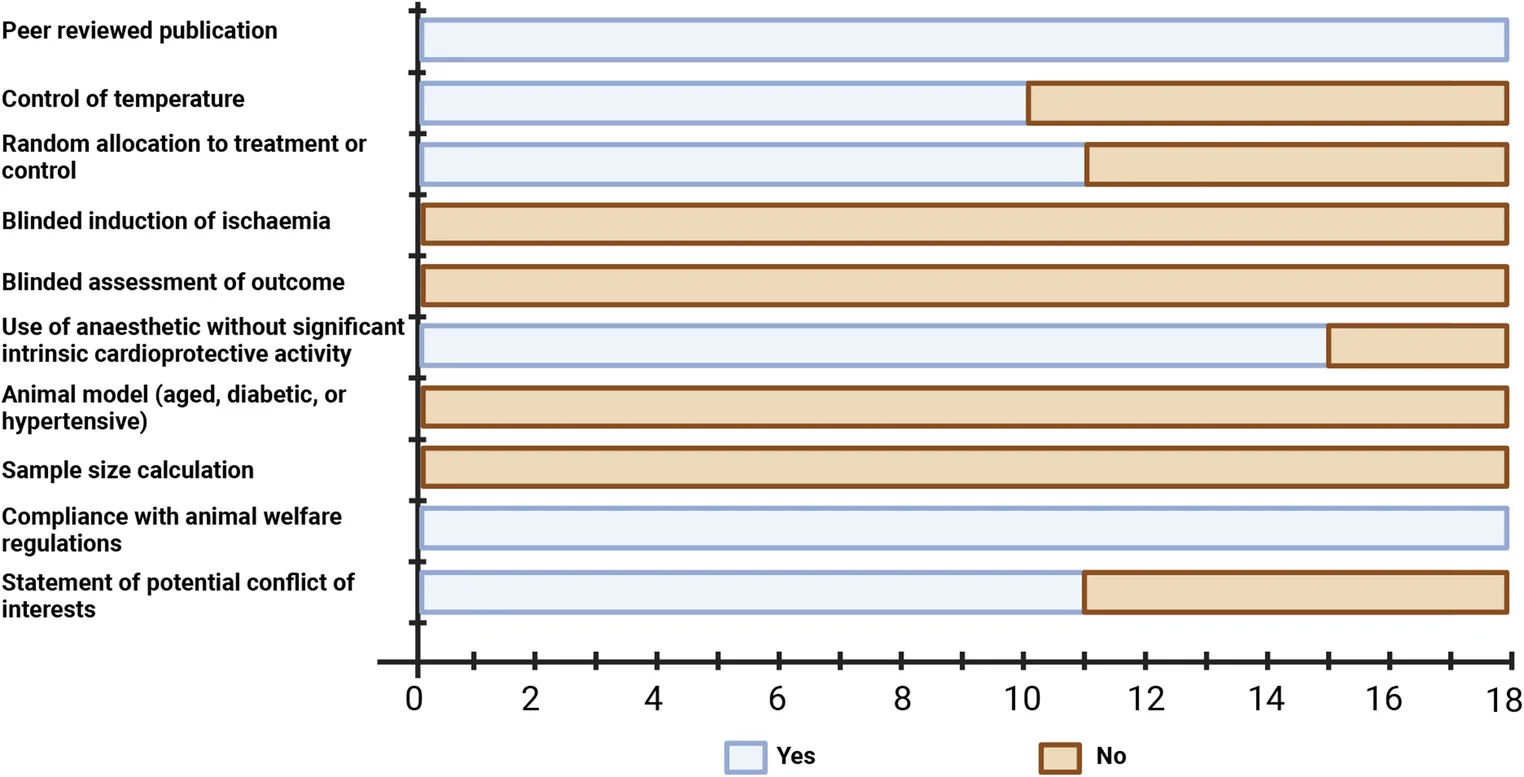
CAMARADES quality assessment outcomes. Overview of quality assessment of 18 papers using the CAMARADES quality assessment tool. Created in BioRender. Ybanez, T. (2026) https://BioRender.com/e417cuk

The CAMARADES quality assessment tool was used to further support the SYRCLE’s risk of bias tool to detect the general quality of reporting in the included papers (Fig. 3). We found that all studies were peer-reviewed, and during the induction of the I/R, only 56% were temperature-controlled. In comparison, 44% either did not mention temperature control or provided inadequate detail about their temperature control protocol. None of the studies made it clear whether the induction of ischaemia during the I/R protocol was blinded; thus, corroborating the findings in SYRCLE’s tool, blinding assessment of the outcomes was unclear in all the studies. All studies used anaesthesia that did not have cardioprotective effects. No chronic treatment studies in animal models, aging, or comorbidity studies were included, and sample size calculations were not mentioned in all papers. Only 61% of studies declared conflicts of interest. However, all studies mentioned adherence to institutional animal ethics.

## Discussion

This systematic review aimed to provide a comprehensive overview of the effects of current pharmacological compounds on mitochondrial and cardiac function in animals exposed to I/R. All 18 studies used male rodents and utilised both ex vivo and in vivo I/R models, with 15 drugs of interest identified. For these studies, the key study outcomes at the end of reperfusion were reported, which will be discussed in subsequent sections and are summarised according to the key primary outcomes: (i) all 15 drugs significantly increased cardiac functional outcomes; (ii) pharmacological compounds reduced infarct size and the levels of cellular injury; and (iii) improved post-ischaemic cardiac function elicited by these pharmacological interventions are due to preserved or augmented markers of mitochondrial function (e.g. mitochondrial respiration, enzyme activity and protein expression).

### Pharmacological Interventions Improve Post-I/R Cardiac Recovery and Prevent Cell Death by Preserving Mitochondrial Respiration and Activity

The outcomes of these studies highlight the importance of a drug’s ability to preserve mitochondrial function in preventing I/R injury. The therapeutic potential of these drugs was also time- and dose-dependent, further complicating the determination of an appropriate treatment protocol to achieve optimal cardioprotection. Nevertheless, all pharmacological interventions prevented left ventricular contractile dysfunction and cell death by modulating various mitochondrial functions, particularly the mitochondrial ETC. Pharmacological compounds improved mitochondrial coupling and enzyme activity, primarily by modulating complex I- and II-driven substrate oxidation, respiration, and protein expression.

Studies also reveal that pharmacological modulation of mitochondrial function can differ between mitochondrial subpopulations. Studies using NaHS and 5-azacytidine revealed that these compounds provided more robust protection against I/R injury in IFM than SSM. At the same time, the pre-ischaemic administration of amobarbital preserved both SSM and IFM complex I function. This discrepancy in the results may be explained by the difference in subpopulation location and their biochemical properties. At rest, the IFM, located between myofibrils, has 1.4 to 1.7 times higher oxidative capacity than SSM, which lies beneath the sarcolemmal membrane [45]. Upon exposure to ischaemia and reperfusion in an isolated perfused rabbit heart, ischaemia reduces the oxidative capacity of the SSM by promoting cardiolipin loss, indicative of structural damage to the mitochondrial inner membrane but not in the IFM [46]. Since cardiolipin is an integral component required for the optimal function of complex IV, the loss of cardiolipin retards oxidative phosphorylation through complex IV and ultimately attenuates SSM activity. Hence, NaHS and 5-azacytidine may not protect the SSM from 30 min of ischaemia at the respective doses tested in the rodent model of I/R injury. Amobarbital may preserve SSM activity by transiently inhibiting electron transport chain activity during ischaemia. This concept will be explored in the next section of this discussion.

Preservation of mitochondrial function could be achieved by leveraging alternative substrates during ischaemia. Hydrogen sulfide from exogenous donors such as NaHS could improve mitochondrial oxidative capacity by utilising sulfide as an alternative substrate, promoting continued ATP production during ischaemia. Due to the bacterial origins of the mitochondria, where sulfide was the primary substrate for energy production of early prokaryotes, it is not surprising that the mitochondria still possess some of their primitive traits [47, 48]. Sulfide was the first inorganic substrate known to be utilised by human cells, and studies have shown that it donates electrons to complex II at lower concentrations [49, 50] and sustains ATP production under oxygen-deficient conditions [51]. However, the use of exogenous hydrogen sulfide should be thoroughly evaluated as concentrations above 20 µM inhibit complex IV activity [52]. Thiosulfate from STS, a major oxidation product of hydrogen sulfide is known to be a safer alternative and could help address this problem.

In contrast, cariporide did not affect mitochondrial respiration when administered alone. This contrasts with the findings of Zhang et al., who found that pre-ischaemic administration of cariporide at a lower concentration (1 µM) reduced myocardial creatine kinase-B and LDH release, suggesting reduced cellular death [53]. It can be speculated that the cardioprotective benefits of cariporide are more pronounced at lower doses, or that the cardioprotective action of NHE-1 inhibitors does not necessarily require modulation of mitochondrial activity. However, a combination of cariporide and metoprolol increased cardiac recovery by increasing mitochondrial oxygen consumption and coupling, associated with decreased mitochondrial calcium overload and reduced mPTP opening. This suggests that the synergistic action of sodium-hydrogen exchanger-1 (NHE-1) and beta-1-adrenergic receptor inhibitors may enhance cardioprotection against I/R injury. Similarly, Angiotensin II alone did not significantly improve mitochondrial respiration despite modestly enhancing cardiac recovery, indicating that its protective effects operate predominantly through extramitochondrial signalling pathways. In contrast, when combined with IPC, Ang II enhanced both cardiac and mitochondrial recovery (complex I) in a PKC-dependent manner. This demonstrates that Ang II does not independently confer mitochondrial benefit but can potentiate established cardioprotective pathways when used as an adjunct.

### Transient and Reversible Pharmacological Inhibition of the Proximal Part of the Mitochondrial Electron Transport Chain is Cardioprotective

A functional mitochondrion at the onset of reperfusion would reduce reperfusion damage and reduce abnormal ROS generation. Therefore, transient mitochondrial shutdown during the ischaemic period would be beneficial for preserving mitochondrial function, followed by gradual reactivation during the reperfusion phase. This has been extensively reviewed elsewhere [54] and corroborates the findings of Chen et al. and Pasdois et al. In the present review, only amobarbital and diazoxide specifically target the electron transport chain complexes. Amobarbital and diazoxide reversibly inhibited complex I and II, respectively, and protected the ETC from ischaemic damage when administered immediately before the ischaemic period [27, 28].

On the other hand, compounds such as 5-azacytidine, NaHS, STS, and DLA preserved or enhanced ETC function during reperfusion and conferred cardioprotection without inhibition of ETC activity during ischaemia. Yang et al. demonstrated that DLA exerted cardioprotection without inhibiting complex I through Sirtuin 1-dependent activation [37]. While these strategies appear to induce contradictory effects on the ETC, the consistent unifying outcome is that the preservation of ETC integrity and oxidative capacity during reperfusion is central to cardioprotection.

The cardioprotective effects of 5-azacytidine, NaHS, STS and DLA likely derive from its pleiotropic effects, which include antioxidant properties and upstream activation of canonical cardioprotective pathway. On the other hand, reversible inhibition of complex I or II during ischaemia by amobarbital and diazoxide prevents pathological ROS generation upon reperfusion by suppressing reverse electron transfer. Inhibition of the ETC may be an attractive strategy for cardioprotection, as it mimics hibernation in animals, in which mitochondria undergo metabolic shutdown without the consequent release of lethal ROS during arousal [55, 56]. This phenomenon is linked to a reduction in the efficiency of complexes I and II during torpor, without a decrease in their protein content, suggesting that post-translational modifications of the ETC may be at play [57]. However, the exact mechanisms governing mitochondrial suppression and reactivation during ischaemia and reperfusion remain incomplete and warrant further investigation.

### Cardioprotective Benefits of Pharmacological Interventions Involve Canonical Cardioprotective Pathways, Regulators of Metabolic Status, and the Induction of Antioxidant Pathways

**Fig. 4:**
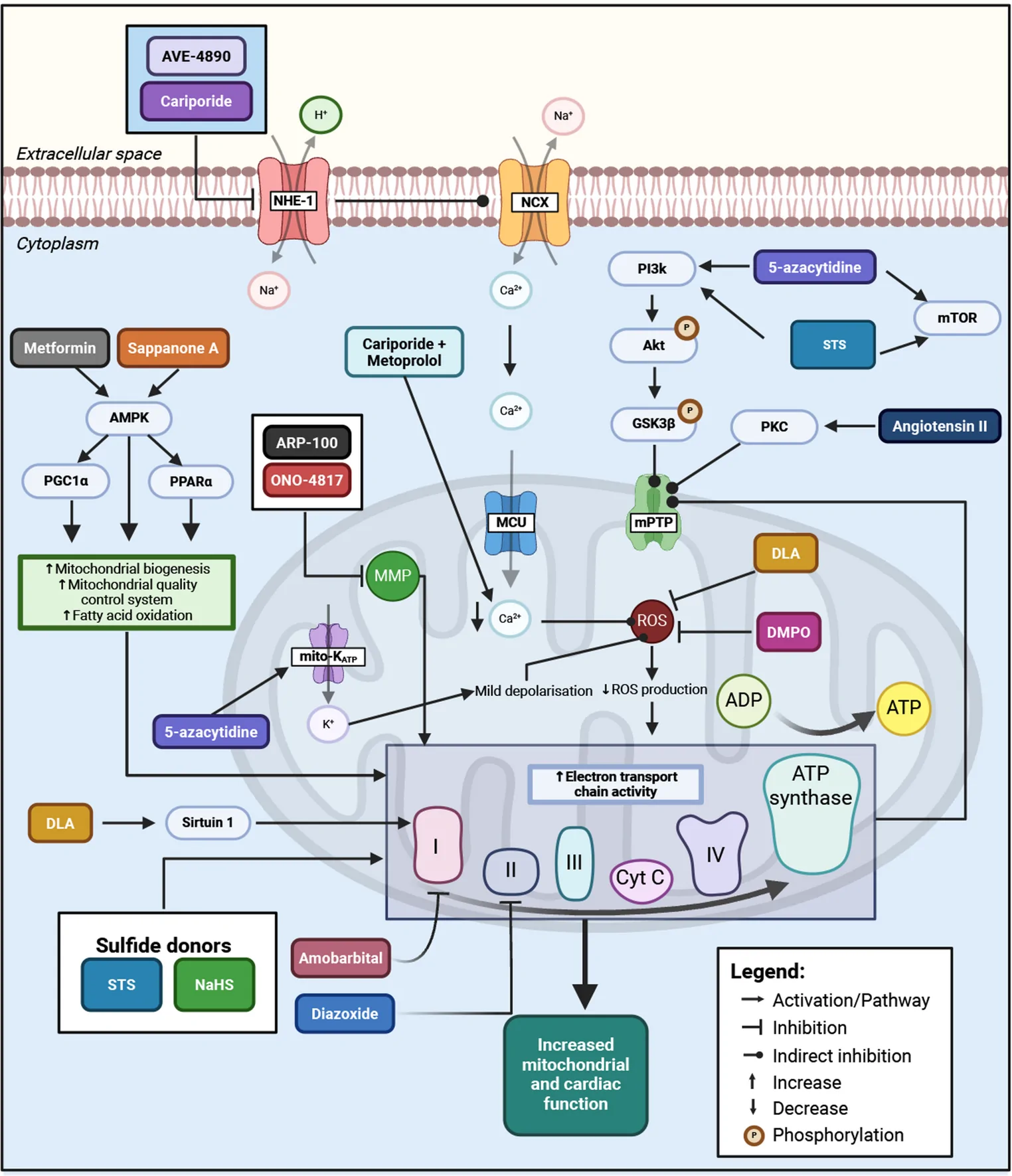
A schematic diagram of molecular pathways induced by pharmacological drugs. Fifteen drugs of interest were investigated, revealing direct and indirect modulation of the mitochondria to preserve cardiac function during ischemia-reperfusion. Created in BioRender. Ybanez, T. (2026) https://BioRender.com/pfdsrtv

The investigation of the effects of pharmacological drugs on mitochondrial function in hearts exposed to I/R confirms that canonical cardioprotective pathways and modulators of cellular metabolic status are involved (Fig. 4). Drugs such as STS and 5-azacytidine activated upstream pathways such as the PI3K–Akt signalling pathway and modulated downstream targets such as GSK3β, mTOR, and the mitoK_ATP_ channel. Activation of this pathway prevents the opening of mPTP, loss of cytochrome c, and caspase activation, among other effects. AVE inhibits NHE-1, which is key for the prevention of Na^+^ accumulation, the reverse activity of the sodium–calcium exchanger (NCX), and subsequent Ca^2+^ accumulation. Angiotensin II promotes cardioprotection via PKC activation, which increases mitochondrial respiration. The cardioprotective mechanisms underlying AVE and Angiotensin II share a common pathway that raises the threshold for mPTP opening. Thus, although pharmacological compounds target distinct canonical pathways, they mimic well-established cardioprotective strategies, such as IPC.

Other upstream mechanisms preserve mitochondrial ETC performance during I/R by modulating mitochondrial parameters outside the internal mitochondrial machinery. These compounds modulate cellular metabolic status and, in turn, affect mitochondrial targets that help maintain mitochondrial homeostasis. For example, induction of the AMPK/PPARα/PGC1α/Sirtuin 1 axis regulates several mitochondrial processes, including mitochondrial bioenergetics, biogenesis, and dynamics, which are part of the mitochondrial quality control system. Administration of STS before ischaemia increased mitochondrial biogenesis via PGC1α activation, leading to increased mitochondrial mRNA copy number for genes related to energy production. Metformin administered in an in vivo or ex vivo I/R protocol and sappanone A activated AMPK, PPARα, and PGC1α – the cell’s metabolic sensors and master regulators of mitochondrial oxidative phosphorylation while also attenuating mitochondrial fission. Sappanone A additionally prevented mitochondrial damage by reducing mitochondrial fragmentation and increasing mitochondrial fusion and mitophagy, thereby maintaining a healthy balance between mitochondrial generation and disposal. Enhanced mitochondrial fusion also improves mitochondrial efficiency by inhibiting MMPs with ARP-100 and ONO-4817. On the other hand, DLA has antioxidant properties associated with Sirtuin 1, while DMPO increases mitochondrial respiration and protein expression by scavenging free radicals. These studies investigated upstream modulators of metabolism and its downstream effects, not just on the mitochondrial ETC but also on the system that maintains it. Understanding the mitochondrial events that support mitochondrial energy production and help preserve ETC function is vital in I/R pathology, as factors that boost mitochondrial oxidative capacity ultimately protect the heart from irreversible damage.

Indeed, recent studies now emphasise quantifying mitochondrial dynamics, mitophagy, mitochondrial biogenesis, and the mitochondrial unfolded protein response, which collectively comprise the mitochondrial quality control (MQC) system [58–62]. Mitochondrial fission and fusion help maintain the structural stability of the ETC and the abundance and quality of the surviving mitochondrial population. Under stress, such as in I/R, mitochondria are highly adaptive and preserve their oxidative capacity by fusing components, including genetic material, proteins, and lipids, between damaged and healthy mitochondria. This prevents mitochondrial membrane potential collapse while maximising oxidative phosphorylation in recycled mitochondria. Mitochondrial fission then segregates dysfunctional mitochondria beyond repair, facilitating their degradation and elimination via mitophagy, a process by which a cell selectively removes impaired mitochondria. Mitochondrial homeostasis is also maintained by the activation of the mitochondrial unfolded protein response during stress-related respiration inhibition, such as in ischaemia, when mitochondrial proteases clear the accumulation of unfolded proteins. In parallel, mitochondrial biogenesis replaces damaged mitochondria by generating new, healthy ones, restarting the cycle.

Recent studies also highlight crosstalk between the endoplasmic reticulum and mitochondria [58], and microtubule–mitochondria interactions [63]. These connections influence calcium homeostasis, MQC regulation, and mitochondrial trafficking, which indirectly support mitochondrial ETC stability and efficiency. This is important because it provides a well-rounded explanation of mitochondrial ETC preservation. A state of equilibrium between mitochondrial turnover and biogenesis, combined with a tightly regulated mitochondrial network and efficient inter-organelle interactions, promotes optimal ETC function, therefore preventing mitochondrial dysfunction and cellular death during I/R. Despite this, we were unable to find papers that met our inclusion criteria beyond 2023, suggesting that recent studies may prioritise quantifying mitochondrial parameters that support oxidative phosphorylation without directly investigating mitochondrial bioenergetics. Future studies may further our understanding of mitochondrial physiology during I/R by examining both indirect and direct markers of mitochondrial function.

Overall, the interactions among molecular pathways induced by pharmacological interventions revealed common endpoints: increased mitochondrial respiration and activity, reduced ROS production, and inhibition of mPTP opening to prevent activation of the cellular death cascade and cardiac dysfunction. While most studies found that ETC preservation following I/R was linked to canonical upstream mechanisms, the absence of other downstream mitochondrial parameters limits our current understanding of mitochondrial bioenergetics during I/R.

### Barriers to Clinical Application of Mitochondria-Targeted Pharmacological Conditioning

The review confirms the view that mitochondria-targeted drugs demonstrate exceptional performance in small animal models. Despite no restrictions on animal species, large animal models did not ultimately meet the inclusion criteria for this review. The lack of direct investigation of mitochondrial parameters, such as mitochondrial respiration and related electron transport chain activities was a key reason for these studies being excluded. This absence of adequate investigation into mitochondrial parameters in larger animal studies is a likely limitation to accurately evaluating drug efficacy and risks overestimating their cardioprotective potential.

During full-text screening, several large animal studies—particularly in pigs—were initially considered but ultimately excluded. This was due to their reliance on surrogate indicators of mitochondrial function, such as membrane potential and ROS levels [64] or limited assessment of mitochondrial respiration (e.g., measuring only one component) [65]. Notably, cyclosporine A (CsA), arguably the most extensively studied mitochondria-targeted drug in both preclinical and clinical settings, was excluded from the pool of papers considered for review. Although it appeared in the initial screening, it was excluded due to the absence of appropriate cardiac endpoints, despite reporting mitochondrial respiration [66].

In addition to differences in animal species and study design, several methodological variables related to mitochondrial outcomes need to be considered. Notably, 16 of the 18 studies used the Langendorff perfusion system, which imposes a substantially lower mechanical workload than isolated working heart preparations, where precise control of afterload and preload is possible and therefore mimics a more physiological response [67]. This difference in energetic demand directly influences mitochondrial function, and thus, the extent of I/R injury and the magnitude of drug-induced effects may differ depending on the perfusion method. Substrate composition in the perfusion buffer must also be considered, particularly the presence of glucose and fatty acids, or their combination, as it can alter mitochondrial substrate utilisation and influence ETC performance, contributing to variability in respiration outcomes [68]. Protocols may also differ in mitochondrial isolation and preparation. Variations in isolation media are known to influence the degree of mitochondrial injury detectable during sample preparation. For example, albumin-containing buffers can stabilise mitochondria by binding free fatty acids and may reduce the extent of damage observed following isolation [69]. Although we did not extract these methodological details across studies, such variability in preparation techniques may introduce additional heterogeneity in mitochondrial outcomes. Finally, temperature control during ischaemia was inconsistently reported, even though slight deviations from normothermia can substantially affect mitochondrial injury.

These factors highlight a significant gap in experimental design and outcome reporting, indicating that the poor clinical translation of cardioprotective therapies is multifactorial, with limited robustness of preclinical I/R models as a key factor [70]. Moreover, the continued preference for surrogate markers suggests an ongoing lack of understanding of mitochondrial pathophysiology in the context of I/R. This knowledge gap hampers progress in drug efficacy testing and delays its clinical application. While some of the drugs reviewed, such as cariporide, diazoxide, metformin, and metoprolol, have advanced to clinical trials, many of these trials either failed to report outcomes, remain incomplete, or were eventually abandoned. Crucially, the endpoints examined were limited to systemic or broad cardiac functional outcomes, with insufficient assessment of mitochondrial function parameters.

These findings point to several persistent issues in the field:

1. A widespread reliance on surrogate rather than direct mitochondrial function measures across experimental stages.
2. Incomplete or no reporting of both mitochondrial and cardiac functional endpoints.
3. A lack of mitochondrial bioenergetic assessment in large animal studies.
4. Limited inclusion of mitochondrial function endpoints in clinical trial design.
5. Inconsistent or missing clinical trial results, or complete abandonment of studies.

Without a robust preclinical foundation, researchers risk underestimating or overestimating a drug’s cardioprotective potential, leading to translational failure and wasted resources. This review highlights a major gap in current preclinical studies that were excluded, where an overreliance on indirect markers continues to hinder the clinical translation of drugs targeting mitochondrial pathways. Quality assessment of the included studies revealed an overall unclear risk of bias, highlighting the need for more rigorous experimental and reporting standards. While the selected papers appropriately focused on direct measurements of mitochondrial outcomes, future investigations should aim for greater transparency and methodological precision to reduce bias and enhance reproducibility. Importantly, despite no restrictions on species and sex, all studies that met the inclusion criteria exclusively involved male rodents. This reveals that the lack of investigation into direct mitochondrial parameters in large animal models and failure to account for sex dependent variability. As a result, current preclinical outcomes are insufficient to delineate how pharmacological agents affect mitochondrial activity at an acute I/R setting. Strengthening these aspects in future research will provide a more reliable preclinical framework and increase the likelihood of successfully translating mitochondria-targeted cardioprotective strategies from bench to bedside.

## Limitations

Several limitations were identified in the studies we evaluated. Firstly, despite not restricting the studies we assessed to rodent models, only investigations using male rodents met al.l the inclusion criteria. Studies involving females and other animal species were excluded during screening based on additional specific criteria. A common motivation for using male rodents is to eliminate data variability caused by the oestrous cycle in females but this has not been shown to be a valid reason for the exclusion of females in I/R studies. Notably, accumulating studies show that mitochondrial responses to I/R is sex-dependent and female hearts exhibited preserved mitochondrial function, reduced oxidative stress and increased tolerance to myocardial tissue damage following I/R, all of which were attributed to estrogen-related cardioprotective effect [71–73]. The effects of pharmacological interventions explored in this review may not reflect the same outcome in female physiology. Hence, future studies must include females to understand the sex-dependent effect on the efficacy of pharmacological compounds in the I/R setting.

Although this review was not restricted to small animals, the criteria excluded studies using larger animal models because mitochondrial parameters, such as oxygen consumption and activity, were not measured. This also suggests that there are very few published investigations assessing cardiac and mitochondrial parameters in larger animal models. This limitation could contribute to the larger problem of the limited translation of the use of these drugs from bench to bedside, as previously discussed.

In addition, due to the complex pathways involved in the chronic effects of pharmacological agents, which require a thorough and separate investigation, chronic studies were excluded to maintain focus on baseline and acute effects. A future systematic evaluation of chronic interventions is warranted and may further strengthen translational understanding. We also limited the inclusion criteria to studies using healthy adult animals without comorbidities and the effect of ageing. This approach aimed to provide a more precise assessment of pharmacological efficacy in the context of I/R injury and to minimise the potential confounding effects of pre-existing conditions. Therefore, a focus on the impacts of pharmacological cardioprotection in aged humans and animals, as well as other risk factors of I/R, is also needed in future reviews. During the screening process, some abstracts mentioned “mitochondrial function” but did not assess or have appropriate outcomes relating to mitochondrial oxidative capacity and enzyme activity, which are critical parameters in the investigation of overall mitochondrial efficiency. In addition, most of the changes in mitochondrial function by the compounds were not due to direct mitochondrial targeting and were secondary to the activation or inhibition of upstream signalling pathways, predominantly the PI3k/Akt pathway. This highlights the challenge of differentiating direct mitochondrial effects from downstream consequences of upstream pathway activation and a limited conclusion about mitochondrial specificity. Hence, a standardised definition of the term “mitochondrial function” is warranted, and a push towards a more direct effect of cardioprotective drugs on mitochondrial activity is necessary to differentiate the impact of upstream mediators on mitochondrial outcomes. Drugs specifically designed to target the different mitochondrial subpopulations are also ideal.

The SYRCLE and CAMARADES quality assessment tools revealed that selection, performance, detection, and reporting biases were evident across papers. Most studies had unclear risk-of-bias scores due to inadequate or vague reporting of the specified items in the tool. Another limitation of this review is that seven studies investigating STS and NaHS were conducted by the same research group or involved overlapping authorships. The use of similar internal protocols within a single research group may affect comparability with studies performed under different methodological conditions. In particular, this review did not conduct a detailed investigation of the components of perfusion buffer or mitochondrial isolation protocols and variability in these methods represents an additional potential source of heterogeneity in the reported cardiac and mitochondrial data. Nevertheless, these were retained as this would significantly reduce the amount of data available for the review. This was also done to ensure consistency of the screening process and prevent selection bias.

It is also important to note that most papers report a positive effect of the drugs on mitochondrial function (except for cariporide or angiotensin II alone) despite current difficulties in translating mitoprotective compounds into the clinical setting. This may be a direct result of inadequate reporting of neutral or negative results, which corroborates the unclear risk of bias across studies. Therefore, the integrity of outcomes could be further improved in future studies through transparent, more detailed reporting of randomisation and blinding methods, reporting all data outcomes (whether positive, neutral, or negative), and acknowledgement of conflicts of interest. Given the various study limitations, the systematic review was structured as a descriptive presentation of results, with no further meta-analysis conducted due to heterogeneity across papers (i.e., differences in units used for several parameters and insufficient data).

## Conclusion

In conclusion, the systematic review of 15 pharmacological compounds used to protect against I/R injury in adult animals showed increased cardiac recovery and infarct tolerance, underpinned by mitochondrial function preservation. Drugs preserved mitochondrial efficiency through various mechanisms, including transient inhibition of the proximal areas of the electron transport chain and modulation of mitochondrial activity via upstream mediators to improve mitochondrial oxidative capacity, enzymatic activity, and protein expression. The involvement of several upstream proteins associated with canonical cardioprotective pathways emphasises the need for future studies to employ a multi-targeted approach in preventing I/R injury. Specifically, a push towards targeted manipulation of components of mitochondrial signalling and function is warranted to inhibit cell death and protect the heart against lethal ischaemia-reperfusion injury.

## Supplementary Information

Below is the link to the electronic supplementary material.

Supplementary Material 1 (DOCX 42.1 KB)

## Data Availability

Data can be obtained from the corresponding author upon reasonable request.

## References

[1] Vaduganathan M, Mensah GA, Turco JV, Fuster V, Roth GA. The global burden of cardiovascular diseases and risk: a compass for future health. J Am Coll Cardiol. 2022;80(25):2361–71. 10.1016/j.jacc.2022.11.005. 36368511

[2] Federation WH. World Heart Report 2023: Confronting the World’s Number One Killer. Geneva, Switzerland. 2023.

[3] Hausenloy DJ, Erik Bøtker H, Condorelli G, et al. Translating cardioprotection for patient benefit: position paper from the Working Group of Cellular Biology of the Heart of the European Society of Cardiology. Cardiovasc Res. 2013;98(1):7–27. 10.1093/cvr/cvt004. 23334258

[4] Jensen RV, Hjortbak MV, Bøtker HE. Ischemic heart disease: an update. Semin Nucl Med. 2020;50(3):195–207. 10.1053/j.semnuclmed.2020.02.007. 32284106

[5] Bentzon JF, Otsuka F, Virmani R, Falk E. Mechanisms of plaque formation and rupture. Circ Res. 2014;114(12):1852–66. 10.1161/circresaha.114.302721. 24902970

[6] Gottlieb RA. Cell death pathways in acute ischemia/reperfusion injury. J Cardiovasc Pharmacol Ther. 2011;16(3–4):233–8. 10.1177/1074248411409581. 21821521 PMC3337030

[7] Peoples JN, Saraf A, Ghazal N, Pham TT, Kwong JQ. Mitochondrial dysfunction and oxidative stress in heart disease. Exp Mol Med. 2019;51(12):1–13. 10.1038/s12276-019-0355-7. 31857574 PMC6923355

[8] Kuznetsov AV, Javadov S, Margreiter R, et al. The role of mitochondria in the mechanisms of cardiac Ischemia-Reperfusion injury. Antioxid (Basel). 2019;8(10). 10.3390/antiox8100454. PMC6826663 31590423

[9] Juhaszova M, Zorov DB, Kim SH, et al. Glycogen synthase kinase-3β mediates convergence of protection signaling to inhibit the mitochondrial permeability transition pore. J Clin Invest. 2004;113(11):1535–49. 10.1172/jci19906. 15173880 PMC419483

[10] Davidson SM, Adameová A, Barile L, et al. Mitochondrial and mitochondrial-independent pathways of myocardial cell death during ischaemia and reperfusion injury. J Cell Mol Med. 2020;24(7):3795–806. 10.1111/jcmm.15127. 32155321 PMC7171390

[11] Murry CE, Jennings RB, Reimer KA. Preconditioning with ischemia: a delay of lethal cell injury in ischemic myocardium. Circulation. 1986;74(5):1124–36. 10.1161/01.cir.74.5.1124. 3769170

[12] Zhao ZQ, Corvera JS, Halkos ME, et al. Inhibition of myocardial injury by ischemic postconditioning during reperfusion: comparison with ischemic preconditioning. Am J Physiol Heart Circ Physiol. 2003;285(2):H579–88. 10.1152/ajpheart.01064.2002. 12860564

[13] Przyklenk K, Bauer B, Ovize M, Kloner RA, Whittaker P. Regional ischemic ‘preconditioning’ protects remote Virgin myocardium from subsequent sustained coronary occlusion. Circulation. 1993;87(3):893–9. 10.1161/01.cir.87.3.893. 7680290

[14] Kharbanda RK, Mortensen UM, White PA, et al. Transient limb ischemia induces remote ischemic preconditioning in vivo. Circulation. 2002;106(23):2881–3. 10.1161/01.cir.0000043806.51912.9b. 12460865

[15] Heusch G. Molecular basis of cardioprotection: signal transduction in ischemic pre-, post-, and remote conditioning. Circ Res. 2015;116(4):674–99. 10.1161/circresaha.116.305348. 25677517

[16] Hausenloy DJ, Yellon DM. Ischaemic conditioning and reperfusion injury. Nat Rev Cardiol. 2016;13(4):193–209. 10.1038/nrcardio.2016.5. 26843289

[17] Shanmuganathan S, Hausenloy DJ, Duchen MR, Yellon DM. Mitochondrial permeability transition pore as a target for cardioprotection in the human heart. Am J Physiol Heart Circ Physiol. 2005;289(1):H237–42. 10.1152/ajpheart.01192.2004. 15961375

[18] Vander Heide RS, Steenbergen C. Cardioprotection and myocardial reperfusion: pitfalls to clinical application. Circ Res. 2013;113(4):464–77. 10.1161/circresaha.113.300765. 23908333 PMC3824252

[19] Heusch G. Critical issues for the translation of cardioprotection. Circ Res. 2017;120(9):1477–86. 10.1161/circresaha.117.310820. 28450365

[20] Page MJ, McKenzie JE, Bossuyt PM, et al. The PRISMA 2020 statement: an updated guideline for reporting systematic reviews. BMJ. 2021;372:n71. 10.1136/bmj.n71. 33782057 PMC8005924

[21] Clark J, Glasziou P, Del Mar C, et al. A full systematic review was completed in 2 weeks using automation tools: a case study. J Clin Epidemiol. 2020;121:81–90. 10.1016/j.jclinepi.2020.01.008. 32004673

[22] Covidence, Melbourne. Australia: Veritas Health Innovation; 2024.

[23] Dutkiewicz T, Chelstowski K. Comparative studies on the influence of decapitation, ketamine and thiopental anesthesia on rat heart mitochondria. Basic Res Cardiol. 1981;76(2):136–43. 10.1007/bf01907952. 7247910

[24] Overmyer KA, Thonusin C, Qi NR, Burant CF, Evans CR. Impact of anesthesia and euthanasia on metabolomics of mammalian tissues: studies in a C57BL/6J mouse model. PLoS ONE. 2015;10(2):e0117232. 10.1371/journal.pone.0117232. 25658945 PMC4319778

[25] Hooijmans CR, Rovers MM, de Vries RB, et al. SYRCLE’s risk of bias tool for animal studies. BMC Med Res Methodol. 2014;14:43. 10.1186/1471-2288-14-43. 24667063 PMC4230647

[26] Macleod MR, O’Collins T, Howells DW, Donnan GA. Pooling of animal experimental data reveals influence of study design and publication bias. Stroke. 2004;35(5):1203–8. 10.1161/01.Str.0000125719.25853.20. 15060322

[27] Chen Q, Moghaddas S, Hoppel CL, Lesnefsky EJ. Reversible blockade of electron transport during ischemia protects mitochondria and decreases myocardial injury following reperfusion. J Pharmacol Exp Ther. 2006;319(3):1405–12. 10.1124/jpet.106.110262. 16990510

[28] Pasdois P, Beauvoit B, Tariosse L, et al. MitoK(ATP)-dependent changes in mitochondrial volume and in complex II activity during ischemic and pharmacological preconditioning of Langendorff-perfused rat heart. J Bioenerg Biomembr. 2006;38(2):101–12. 10.1007/s10863-006-9016-3. 17031549

[29] Ravindran S, Ansari Banu S, Kurian GA. Hydrogen sulfide preconditioning shows differential protection towards interfibrillar and subsarcolemmal mitochondria from isolated rat heart subjected to revascularization injury. Cardiovasc Pathol. 2016;25(4):306–15. 10.1016/j.carpath.2016.04.005. 27167777

[30] Banu SA, Ravindran S, Kurian GA. Hydrogen sulfide post-conditioning preserves interfibrillar mitochondria of rat heart during ischemia reperfusion injury. Cell Stress Chaperones. 2016;21(4):571–82. 10.1007/s12192-016-0682-8. 26951457 PMC4907988

[31] Ravindran S, Kurian GA. Preconditioning the rat heart with sodium thiosulfate preserved the mitochondria in response to ischemia-reperfusion injury. J Bioenerg Biomembr. 2019;51(3):189–201. 10.1007/s10863-019-09794-8. 30929125

[32] Kannan S, Boovarahan SR, Rengaraju J, Prem P, Kurian GA. Attenuation of cardiac ischemia-reperfusion injury by sodium thiosulfate is partially dependent on the effect of cystathione beta synthase in the myocardium. Cell Biochem Biophys. 2019;77(3):261–72. 10.1007/s12013-019-00871-8. 31065867

[33] Boovarahan SR, Venkatasubramanian H, Sharma N, et al. Inhibition of PI3K/mTOR/K(ATP) channel blunts sodium thiosulphate preconditioning mediated cardioprotection against ischemia-reperfusion injury. Arch Pharm Res. 2021;44(6):605–20. 10.1007/s12272-021-01339-1. 34170496

[34] Ravindran S, Jahir Hussain S, Boovarahan SR, Kurian GA. Sodium thiosulfate post-conditioning protects rat hearts against ischemia reperfusion injury via reduction of apoptosis and oxidative stress. Chem Biol Interact. 2017;274:24–34. 10.1016/j.cbi.2017.07.002. 28688941

[35] Javadov S, Choi A, Rajapurohitam V, et al. NHE-1 inhibition-induced cardioprotection against ischaemia/reperfusion is associated with attenuation of the mitochondrial permeability transition. Cardiovasc Res. 2008;77(2):416–24. 10.1093/cvr/cvm039. 18006455

[36] Wang P, Zaragoza C, Holman W. Sodium-hydrogen exchange inhibition and beta-blockade additively decrease infarct size. Ann Thorac Surg. 2007;83(3):1121–7. 10.1016/j.athoracsur.2006.10.039. 17307470

[37] Yang XY, He K, Pan CS, et al. 3, 4-dihydroxyl-phenyl lactic acid restores NADH dehydrogenase 1 α subunit 10 to ameliorate cardiac reperfusion injury. Sci Rep. 2015;5:10739. 10.1038/srep10739. 26030156 PMC5377067

[38] Zuo L, Chen YR, Reyes LA, et al. The radical trap 5,5-dimethyl-1-pyrroline N-oxide exerts dose-dependent protection against myocardial ischemia-reperfusion injury through preservation of mitochondrial electron transport. J Pharmacol Exp Ther. 2009;329(2):515–23. 10.1124/jpet.108.143479. 19201989 PMC2672876

[39] Bassiouni W, Valencia R, Mahmud Z, Seubert JM, Schulz R. Matrix metalloproteinase-2 proteolyzes mitofusin-2 and impairs mitochondrial function during myocardial ischemia-reperfusion injury. Basic Res Cardiol. 2023;118(1):29. 10.1007/s00395-023-00999-y. 37495895

[40] Barreto-Torres G, Parodi-Rullán R, Javadov S. The role of PPARα in metformin-induced attenuation of mitochondrial dysfunction in acute cardiac ischemia/reperfusion in rats. Int J Mol Sci. 2012;13(6):7694–709. 10.3390/ijms13067694. 22837722 PMC3397554

[41] Palee S, Higgins L, Leech T, Chattipakorn SC, Chattipakorn N. Acute Metformin treatment provides cardioprotection via improved mitochondrial function in cardiac ischemia / reperfusion injury. Biomed Pharmacother. 2020;130:110604. 10.1016/j.biopha.2020.110604. 32777704

[42] Shi X, Li Y, Wang Y, et al. Pharmacological postconditioning with Sappanone A ameliorates myocardial ischemia reperfusion injury and mitochondrial dysfunction via AMPK-mediated mitochondrial quality control. Toxicol Appl Pharmacol. 2021;427:115668. 10.1016/j.taap.2021.115668. 34358556

[43] Boovarahan SR, Kurian GA. Preconditioning the rat heart with 5-azacytidine attenuates myocardial ischemia/reperfusion injury via PI3K/GSK3β and mitochondrial K(ATP) signaling axis. J Biochem Mol Toxicol. 2021;35(12):e22911. 10.1002/jbt.22911. 34462995

[44] Nuñez RE, Castro M, Javadov S, Escobales N. Angiotensin II and ischemic preconditioning synergize to improve mitochondrial function while showing additive effects on ventricular postischemic recovery. J Cardiovasc Pharmacol. 2014;64(2):172–9. 10.1097/fjc.0000000000000103. 24705171 PMC4126876

[45] Palmer JW, Tandler B, Hoppel CL. Biochemical properties of subsarcolemmal and interfibrillar mitochondria isolated from rat cardiac muscle. J Biol Chem. 1977;252(23):8731–9. 925018

[46] Lesnefsky EJ, Slabe TJ, Stoll MS, Minkler PE, Hoppel CL. Myocardial ischemia selectively depletes Cardiolipin in rabbit heart subsarcolemmal mitochondria. Am J Physiol Heart Circ Physiol. 2001;280(6):H2770–8. 10.1152/ajpheart.2001.280.6.H2770. 11356635

[47] Olson KR. Mitochondrial adaptations to utilize hydrogen sulfide for energy and signaling. J Comp Physiol B. 2012;182(7):881–97. 10.1007/s00360-012-0654-y. 22430869

[48] Olson KR, Straub KD. The role of hydrogen sulfide in evolution and the evolution of hydrogen sulfide in metabolism and signaling. Physiol (Bethesda). 2016;31(1):60–72. 10.1152/physiol.00024.2015. 26674552

[49] Módis K, Panopoulos P, Coletta C, Papapetropoulos A, Szabo C. Hydrogen sulfide-mediated stimulation of mitochondrial electron transport involves inhibition of the mitochondrial phosphodiesterase 2A, elevation of cAMP and activation of protein kinase A. Biochem Pharmacol. 2013;86(9):1311–9. 10.1016/j.bcp.2013.08.064. 24012591

[50] Szabo C. Hydrogen Sulfide, an endogenous stimulator of mitochondrial function in cancer cells. Cells. 2021;10(2). 10.3390/cells10020220. PMC7911547 33499368

[51] Fu M, Zhang W, Wu L, et al. Hydrogen sulfide (H_2_S) metabolism in mitochondria and its regulatory role in energy production. Proc Natl Acad Sci U S A. 2012;109(8):2943–8. 10.1073/pnas.1115634109. 22323590 PMC3287003

[52] Bouillaud F, Blachier F. Mitochondria and sulfide: a very old story of poisoning, feeding, and signaling? Antioxid Redox Signal. 2011;15(2):379–91. 10.1089/ars.2010.3678. 21028947

[53] Zhang Y, Chen J, Zhang F, Xia Q. Cariporide attenuates myocardial ischaemia, reperfusion injury and apoptosis in isolated rat hearts. Acta Cardiol. 2006;61(6):637–41. 10.2143/ac.61.6.2017963. 17205921

[54] Burwell LS, Nadtochiy SM, Brookes PS. Cardioprotection by metabolic shut-down and gradual wake-up. J Mol Cell Cardiol. 2009;46(6):804–10. 10.1016/j.yjmcc.2009.02.026. 19285082 PMC2683198

[55] Muleme HM, Walpole AC, Staples JF. Mitochondrial metabolism in hibernation: metabolic suppression, temperature effects, and substrate preferences. Physiol Biochem Zool. 2006;79(3):474–83. 10.1086/501053. 16691514

[56] Brown JC, Chung DJ, Belgrave KR, Staples JF. Mitochondrial metabolic suppression and reactive oxygen species production in liver and skeletal muscle of hibernating thirteen-lined ground squirrels. Am J Physiol Regul Integr Comp Physiol. 2012;302(1):R15–28. 10.1152/ajpregu.00230.2011. 21993528

[57] Mathers KE, McFarlane SV, Zhao L, Staples JF. Regulation of mitochondrial metabolism during hibernation by reversible suppression of electron transport system enzymes. J Comp Physiol B. 2017;187(1):227–34. 10.1007/s00360-016-1022-0. 27497598

[58] Wang J, Zhuang H, Li C, et al. Ligustrazine nano-drug delivery system ameliorates doxorubicin-mediated myocardial injury via piezo-type mechanosensitive ion channel component 1-prohibitin 2-mediated mitochondrial quality surveillance. J Nanobiotechnol. 2025;23(1):383. 10.1186/s12951-025-03420-z. PMC12117932 40426179

[59] Pu X, Wu Q, Yan Z, et al. Tanshinone IIA modulates Sirt5 and Metll3 interaction to govern mitochondria-endoplasmic reticulum unfolded protein response in coronary microvascular injury. Phytomedicine. 2025;145:156982. 10.1016/j.phymed.2025.156982. 40543233

[60] Chang X, Zhou S, Huang Y, et al. Zishen huoxue decoction (ZSHX) alleviates ischemic myocardial injury (MI) via Sirt5-β-tubulin mediated synergistic mechanism of “mitophagy-unfolded protein response” and mitophagy. Chin J Nat Med. 2025;23(3):311–21. 10.1016/s1875-5364(25)60838-7. 40122661

[61] Chang X, Zhou H, Hu J, et al. Targeting mitochondria by lipid-selenium conjugate drug results in malate/fumarate exhaustion and induces mitophagy-mediated necroptosis suppression. Int J Biol Sci. 2024;20(14):5793–811. 10.7150/ijbs.102424. 39494338 PMC11528455

[62] Donner DG, Kong AM, Lees JG, et al. Acute inhibition of Drp1 mitigates adverse cardiac remodelling following chronic reperfused myocardial infarction. Cardiovasc Drugs Ther. 2025;39(5):953–9. 10.1007/s10557-024-07633-6. 39432235

[63] Wu Q, Wang Y, Liu J, et al. Microtubules and cardiovascular diseases: insights into pathology and therapeutic strategies. Int J Biochem Cell Biol. 2024;175:106650. 10.1016/j.biocel.2024.106650. 39237031

[64] Palee S, Weerateerangkul P, Surinkeaw S, Chattipakorn S, Chattipakorn N. Effect of Rosiglitazone on cardiac electrophysiology, infarct size and mitochondrial function in ischaemia and reperfusion of swine and rat heart. Exp Physiol. 2011;96(8):778–89. 10.1113/expphysiol.2011.057885. 21666037

[65] Kolseth SM, Wahba A, Kirkeby-Garstad I, et al. A dose–response study of levosimendan in a porcine model of acute ischaemic heart failure. Eur J Cardiothorac Surg. 2011;41(6):1377–83. 10.1093/ejcts/ezr201. 22219475

[66] Gedik N, Heusch G, Skyschally A. Infarct size reduction by cyclosporine A at reperfusion involves inhibition of the mitochondrial permeability transition pore but does not improve mitochondrial respiration. Arch Med Sci. 2013;9(6):968–75. 10.5114/aoms.2013.38175. 24482638 PMC3902704

[67] Usai DS, Aasum E, Thomsen MB. The isolated, perfused working heart Preparation of the mouse-Advantages and pitfalls. Acta Physiol (Oxf). 2025;241(4):e70023. 10.1111/apha.70023. 40078031 PMC11904386

[68] Belke DD, Larsen TS, Lopaschuk GD, Severson DL. Glucose and fatty acid metabolism in the isolated working mouse heart. Am J Physiol. 1999;277(4):R1210–7. 10.1152/ajpregu.1999.277.4.R1210. 10516264

[69] Panov AV, Vavilin VA, Lyakhovich VV, Brooks BR, Bonkovsky HL. Effect of bovine serum albumin on mitochondrial respiration in the brain and liver of mice and rats. Bull Exp Biol Med. 2010;149(2):187–90. 10.1007/s10517-010-0904-5. 21113488

[70] Heusch G. Myocardial ischemia/reperfusion: translational pathophysiology of ischemic heart disease. Med. 2024;5(1):10–31. 10.1016/j.medj.2023.12.007. 38218174

[71] Murphy E, Steenbergen C. Gender-based differences in mechanisms of protection in myocardial ischemia-reperfusion injury. Cardiovasc Res. 2007;75(3):478–86. 10.1016/j.cardiores.2007.03.025. 17466956

[72] Schubert C, Raparelli V, Westphal C, et al. Reduction of apoptosis and preservation of mitochondrial integrity under ischemia/reperfusion injury is mediated by estrogen receptor β. Biol Sex Differ. 2016;7:53. 10.1186/s13293-016-0104-8. 27688871 PMC5035458

[73] Scott SR, Singh K, Yu Q, Sen CK, Wang M. Sex as biological variable in cardiac mitochondrial bioenergetic responses to acute stress. Int J Mol Sci. 2022;23(16). 10.3390/ijms23169312. PMC9409303 36012574

