# Supplementary material for: Cardioprotection Through Mitochondrial Modulation: A Systematic Review of Pharmacological Interventions in Animal Models of I/R Injury
Source: Cardiovasc Drugs Ther. 2026 Mar 4;40(5):1861–80. doi: 10.1007/s10557-026-07851-0 (PMC13578120; doi:10.1007/s10557-026-07851-0)
Supplement: Supplementary file 1 [file 10557_2026_7851_MOESM1_ESM.docx]

**Cardioprotection Through Mitochondrial Modulation: A Systematic Review of Pharmacological Interventions in Animal Models of I/R Injury**

Trissha Ybanez^1^ (ORCID: [0000-0002-4448-9026](https://orcid.org/0000-0002-4448-9026)), Joshua Ingles^2^ (ORCID: [0000-0001-8375-7305](https://orcid.org/0000-0001-8375-7305)), Eugene Du Toit^1^ (ORCID: [0000-0002-5588-2421](https://orcid.org/0000-0002-5588-2421)) Jason Peart^1^ (ORCID: [0000-0002-1027-9995](https://orcid.org/0000-0002-1027-9995))

^1^*School of Pharmacy and Medical Science, Griffith University, Southport, Queensland, 4217, Australia;*

^2^*Institute for Biomedicine and Glycomics, Griffith University, Southport, Queensland 4215, Australia*

**Corresponding author:**

Trissha Ybanez

School of Pharmacy and Medical Science,

Griffith University, Southport 4217 QLD

AUSTRALIA

***Email:*** [trissha.ybanez@griffithuni.edu.au](mailto:trissha.ybanez@griffithuni.edu.au) OR [t.ybanez@griffith.edu.au](mailto:t.ybanez@griffith.edu.au)

**Supplementary Information (SI)**

**SI 1 - PRISMA 2020 checklist**

| **Section and Topic** | **Item #** | **Checklist item** | **Location where item is reported** |
| --- | --- | --- | --- |
| **TITLE** | | |  |
| Title | 1 | Identify the report as a systematic review. | First heading |
| **ABSTRACT** | | |  |
| Abstract | 2 | See the PRISMA 2020 for Abstracts checklist. | Abstract section |
| **INTRODUCTION** | | |  |
| Rationale | 3 | Describe the rationale for the review in the context of existing knowledge. | Section 1 |
| Objectives | 4 | Provide an explicit statement of the objective(s) or question(s) the review addresses. | Section 1 |
| **METHODS** | | |  |
| Eligibility criteria | 5 | Specify the inclusion and exclusion criteria for the review and how studies were grouped for the syntheses. | Sections 2.3. and 2.4. |
| Information sources | 6 | Specify all databases, registers, websites, organisations, reference lists and other sources searched or consulted to identify studies. Specify the date when each source was last searched or consulted. | Section 2.1. |
| Search strategy | 7 | Present the full search strategies for all databases, registers and websites, including any filters and limits used. | Section 2 |
| Selection process | 8 | Specify the methods used to decide whether a study met the inclusion criteria of the review, including how many reviewers screened each record and each report retrieved, whether they worked independently, and if applicable, details of automation tools used in the process. | Section 2 |
| Data collection process | 9 | Specify the methods used to collect data from reports, including how many reviewers collected data from each report, whether they worked independently, any processes for obtaining or confirming data from study investigators, and if applicable, details of automation tools used in the process. | Section 2 |
| Data items | 10a | List and define all outcomes for which data were sought. Specify whether all results that were compatible with each outcome domain in each study were sought (e.g. for all measures, time points, analyses), and if not, the methods used to decide which results to collect. | Section 2 |
|  | 10b | List and define all other variables for which data were sought (e.g. participant and intervention characteristics, funding sources). Describe any assumptions made about any missing or unclear information. | Section 2 |
| Study risk of bias assessment | 11 | Specify the methods used to assess risk of bias in the included studies, including details of the tool(s) used, how many reviewers assessed each study and whether they worked independently, and if applicable, details of automation tools used in the process. | Section 2 |
| Effect measures | 12 | Specify for each outcome the effect measure(s) (e.g. risk ratio, mean difference) used in the synthesis or presentation of results. | Not reported |
| Synthesis methods | 13a | Describe the processes used to decide which studies were eligible for each synthesis (e.g. tabulating the study intervention characteristics and comparing against the planned groups for each synthesis (item #5)). | Section 2 |
|  | 13b | Describe any methods required to prepare the data for presentation or synthesis, such as handling of missing summary statistics, or data conversions. | Section 2 |
|  | 13c | Describe any methods used to tabulate or visually display results of individual studies and syntheses. | Section 2 |
|  | 13d | Describe any methods used to synthesize results and provide a rationale for the choice(s). If meta-analysis was performed, describe the model(s), method(s) to identify the presence and extent of statistical heterogeneity, and software package(s) used. | Section 2 |
|  | 13e | Describe any methods used to explore possible causes of heterogeneity among study results (e.g. subgroup analysis, meta-regression). | Not reported. |
|  | 13f | Describe any sensitivity analyses conducted to assess robustness of the synthesized results. | Not reported. |
| Reporting bias assessment | 14 | Describe any methods used to assess risk of bias due to missing results in a synthesis (arising from reporting biases). | Section 2.5. |
| Certainty assessment | 15 | Describe any methods used to assess certainty (or confidence) in the body of evidence for an outcome. | Not reported. |
| **RESULTS** | | |  |
| Study selection | 16a | Describe the results of the search and selection process, from the number of records identified in the search to the number of studies included in the review, ideally using a flow diagram. | Section 3.1 |
|  | 16b | Cite studies that might appear to meet the inclusion criteria, but which were excluded, and explain why they were excluded. | Section 3.2 |
| Study characteristics | 17 | Cite each included study and present its characteristics. | Each study is cited appropriately across section 3, and in Tables 2 and 3 |
| Risk of bias in studies | 18 | Present assessments of risk of bias for each included study. | Section 3 and Figure 2 and 3 |
| Results of individual studies | 19 | For all outcomes, present, for each study: (a) summary statistics for each group (where appropriate) and (b) an effect estimate and its precision (e.g. confidence/credible interval), ideally using structured tables or plots. | Summary of descriptive statistics were reported in Table 2 and 3 |
| Results of syntheses | 20a | For each synthesis, briefly summarise the characteristics and risk of bias among contributing studies. | Risk of bias is discussed descriptively in section 4 |
|  | 20b | Present results of all statistical syntheses conducted. If meta-analysis was done, present for each the summary estimate and its precision (e.g. confidence/credible interval) and measures of statistical heterogeneity. If comparing groups, describe the direction of the effect. | Not reported. |
|  | 20c | Present results of all investigations of possible causes of heterogeneity among study results. | Not reported. |
|  | 20d | Present results of all sensitivity analyses conducted to assess the robustness of the synthesized results. | Not reported. |
| Reporting biases | 21 | Present assessments of risk of bias due to missing results (arising from reporting biases) for each synthesis assessed. | Section 4 |
| Certainty of evidence | 22 | Present assessments of certainty (or confidence) in the body of evidence for each outcome assessed. | Not reported |
| **DISCUSSION** | | |  |
| Discussion | 23a | Provide a general interpretation of the results in the context of other evidence. | Section 5 |
|  | 23b | Discuss any limitations of the evidence included in the review. | Section 6 |
|  | 23c | Discuss any limitations of the review processes used. | Section 6 |
|  | 23d | Discuss implications of the results for practice, policy, and future research. | Section 6 |
| **OTHER INFORMATION** | | |  |
| Registration and protocol | 24a | Provide registration information for the review, including register name and registration number, or state that the review was not registered. | The review was not registered. |
|  | 24b | Indicate where the review protocol can be accessed, or state that a protocol was not prepared. | N/A |
|  | 24c | Describe and explain any amendments to information provided at registration or in the protocol. | N/A |
| Support | 25 | Describe sources of financial or non-financial support for the review, and the role of the funders or sponsors in the review. | Section 9 |
| Competing interests | 26 | Declare any competing interests of review authors. | Section 9 |
| Availability of data, code and other materials | 27 | Report which of the following are publicly available and where they can be found: template data collection forms; data extracted from included studies; data used for all analyses; analytic code; any other materials used in the review. | All data are available within the text or in the supplementary material. |

*From:*  Page MJ, McKenzie JE, Bossuyt PM, Boutron I, Hoffmann TC, Mulrow CD, et al. The PRISMA 2020 statement: an updated guideline for reporting systematic reviews. BMJ 2021;372:n71. doi: 10.1136/bmj.n71

**SI 2 – List of search strings for each database**

**PubMed (Advanced search):**

("Myocardial reperfusion injury"[MeSH Terms:noexp] OR "myocardial ischaemia"[Title/Abstract] OR "myocardial ischemia"[Title/Abstract] OR "ischaemia/reperfusion injury"[Title/Abstract] OR "ischemia/reperfusion injury"[Title/Abstract] OR "ischemia reperfusion"[Title/Abstract] OR "ischaemia reperfusion"[Title/Abstract] OR "myocardial ischaemia reperfusion injury"[Title/Abstract] OR "myocardial ischemia reperfusion injury"[Title/Abstract] OR "ischemi*"[Title/Abstract] OR "ischaemi*"[Title/Abstract] OR "reperfusion"[Title/Abstract] OR "ischemia/reperfusion injury"[Title/Abstract] OR "ischaemia/reperfusion injury"[Title/Abstract]) AND ("cardiac"[Title/Abstract] OR "heart*"[Title/Abstract] OR "myocardi*"[Title/Abstract]) AND ("mitochondri*"[Title/Abstract] OR "mitochondria, heart"[MeSH Terms:noexp] OR "cardiac mitochondri*"[Title/Abstract]) AND ("animals"[MeSH Terms:noexp] OR "animal model*"[Title/Abstract] OR "disease models, animal"[MeSH Terms:noexp]) AND ("mitochondrial function"[Title/Abstract] OR "mitochondrial respiration"[Title/Abstract] OR "mitochondrial oxygen consumption"[Title/Abstract]) AND ((("drug therapy"[Text Word] OR "pharmacolog*"[Text Word] OR "pharmacology"[MeSH Terms:noexp] OR "drug*"[Text Word] OR "drug effect*"[Text Word]) AND "protective agents"[MeSH Terms:noexp]) OR "agonist*"[Text Word] OR "antagonist*"[Text Word] OR "anti inflammatory agents*"[MeSH Terms:noexp] OR "cardiotonic agents"[MeSH Terms:noexp] OR "inhibitor*"[Text Word] OR "activator*"[Text Word])

**Embase (Elsevier; Advanced search):**

('myocardial reperfusion injury'/de OR 'myocardial ischaemia':ti,ab OR 'myocardial ischemia':ti,ab OR 'ischaemia/reperfusion injury':ti,ab OR 'ischemia/reperfusion injury':ti,ab OR 'ischemia'/de OR 'ischaemia'/de OR 'myocardial ischaemia reperfusion injury':ti,ab OR 'myocardial ischemia reperfusion injury':ti,ab OR ischemi*:ti,ab OR ischaemi*:ti,ab OR reperfusion:ti,ab OR 'ischemia reperfusion injury':ti,ab OR 'ischaemia reperfusion injury':ti,ab) AND (cardiac:ti,ab OR heart*:ti,ab OR myocardi*:ti,ab) AND (mitochondri*:ti,ab OR 'mitochondria, heart'/de OR 'cardiac mitochondri*':ti,ab) AND ('animals'/de OR 'animal model*':ti,ab OR 'disease models, animal'/de) AND ('mitochondrial function':ti,ab OR 'mitochondrial respiration':ti,ab OR 'mitochondrial oxygen consumption':ti,ab) AND ('drug therapy' OR pharmacolog* OR 'pharmacology'/de OR drug* OR 'drug effect' OR 'protective agent'/de OR agonist* OR antagonist* OR 'anti-inflammatory agents*' OR 'cardiotonic agents'/de OR inhibitor* OR activator*)

**Scopus (Advanced search):**

( INDEXTERMS ( "Myocardial reperfusion injury" )  OR  TITLE-ABS ( "myocardial ischaemia" )  OR  TITLE-ABS ( "myocardial ischemia" )  OR  TITLE-ABS ( "ischaemia/reperfusion injury" )  OR  TITLE-ABS ( "ischemia/reperfusion injury" )  OR  INDEXTERMS ( ischemia )  OR  INDEXTERMS ( ischaemia )  OR  TITLE-ABS ( "myocardial ischaemia reperfusion injury" )  OR  TITLE-ABS ( "myocardial ischemia reperfusion injury" )  OR  TITLE-ABS ( ischemi* )  OR  TITLE-ABS ( ischaemi* )  OR  TITLE-ABS ( reperfusion )  OR  TITLE-ABS ( "ischemia reperfusion injury" )  OR  TITLE-ABS ( "ischaemia reperfusion injury" ) )  AND  ( TITLE-ABS ( cardiac )  OR  TITLE-ABS ( heart* )  OR  TITLE-ABS ( myocardi* ) )  AND  ( TITLE-ABS ( mitochondri* )  OR  INDEXTERMS ( "mitochondria, heart" )  OR  TITLE-ABS ( "cardiac mitochondri*" ) )  AND  ( INDEXTERMS ( animals )  OR  TITLE-ABS ( "animal model*" )  OR  INDEXTERMS ( "disease models, animal" ) )  AND  ( TITLE-ABS ( "mitochondrial function" )  OR  TITLE-ABS ( "mitochondrial respiration" )  OR  TITLE-ABS ( "mitochondrial oxygen consumption" ) )  AND  ( TITLE-ABS-KEY ( "drug therapy" )  OR  TITLE-ABS-KEY ( pharmacolog* )  OR  INDEXTERMS ( pharmacology )  OR  TITLE-ABS-KEY ( drug* )  OR  INDEXTERMS ( "drug effect* "  protective  AND agents  "" )  OR  TITLE-ABS-KEY ( agonist* )  OR  TITLE-ABS-KEY ( antagonist* )  OR  INDEXTERMS ( "anti-inflammatory agents*" )  OR  INDEXTERMS ( "cardiotonic agents" )  OR  TITLE-ABS-KEY ( inhibitor* )  OR  TITLE-ABS-KEY ( activator* ) )

**Web of Science (Advanced search):**

#1

ALL="Myocardial reperfusion injury" OR (TI="myocardial ischaemia" OR AB="myocardial ischaemia") OR (TI="myocardial ischemia" OR AB="myocardial ischemia") OR (TI="ischaemia/reperfusion injury" OR AB="ischaemia/reperfusion injury") OR (TI="ischemia/reperfusion injury" OR AB="ischemia/reperfusion injury") OR ALL=ischemia OR ALL=ischaemia OR (TI="myocardial ischaemia reperfusion injury" OR AB="myocardial ischaemia reperfusion injury") OR (TI="myocardial ischemia reperfusion injury" OR AB="myocardial ischemia reperfusion injury") OR (TI=ischemi* OR AB=ischemi*) OR (TI=ischaemi* OR AB=ischaemi*) OR (TI=reperfusion OR AB=reperfusion) OR (TI="ischemia reperfusion injury" OR AB="ischemia reperfusion injury") OR (TI="ischaemia reperfusion injury" OR AB="ischaemia reperfusion injury")

#2

(TI=cardiac OR AB=cardiac) OR (TI=heart* OR AB=heart*) OR (TI=myocardi* OR AB=myocardi*)

#3

(TI=mitochondri* OR AB=mitochondri*) OR ALL="mitochondria, heart" OR (TI="cardiacmitochondri*" OR AB="cardiac mitochondri*")

#4

ALL=animals OR (TI="animal model*" OR AB="animal model*") OR ALL="disease models, animal"

#5

(TI="mitochondrial function" OR AB="mitochondrial function") OR (TI="mitochondrial respiration" OR AB="mitochondrial respiration") OR (TI="mitochondrial oxygen consumption" OR AB="mitochondrial oxygen consumption")

#6

ALL="drug therapy" OR ALL=pharmacolog* OR ALL=Pharmacology OR ALL=drug* OR ALL=agonist* OR ALL=antagonist* OR ALL="anti-inflammatory agents*" OR ALL="cardiotonic agents" OR ALL=inhibitor* OR ALL=Activator*

#7

Combine search queries: #1 AND #2 AND #3 AND #4 AND #5 AND #6

**SI 3 – Study characteristics and I/R protocol**

| **First author** | **Year** | **Gender** | **Animal** | **Type of I/R (*In vivo*/*Ex vivo*)** | **IR method** | **Duration of Ischaemia (min)** | **Duration of Reperfusion (min)** | **Ref.** |
| --- | --- | --- | --- | --- | --- | --- | --- | --- |
| **Banu et al.** | **2016** | Male | Wistar rats | *Ex vivo* | Isolated heart perfusion (Langendorff method) | 30 min | 60 min | [30] |
| **Barreto-Torres et al.** | **2012** | Male | Sprague-Dawley rats | *Ex vivo* | Isolated heart perfusion (Langendorff method) | 30 min | 30 min | [40] |
| **Bassouini et al.** | **2023** | Male | C57BL/6J mice | *Ex vivo* | Isolated heart perfusion (Langendorff method) | 45 min | 120 min | [39] |
| **Boovarahan & Kurian** | **2021** | Male | Wistar rats | *Ex vivo* | Isolated heart perfusion (Langendorff method) | 30 min | 60 min | [43] |
| **Boovarahan et al.** | **2021** | Male | Wistar rats | *Ex vivo* | Isolated heart perfusion (Langendorff method) | 30 min | 60 min | [31] |
| **Chen et al.** | **2006** | Male | Fischer-344 rats | *Ex vivo* | Isolated heart perfusion (Langendorff method) | 25 min | 30 min | [27] |
| **Javadov et al.** | **2008** | Male | Sprague-Dawley rats | *Ex vivo* | Isolated heart perfusion (Langendorff method) | 40 min | 60 min | [35] |
| **Kannan et al.** | **2019** | Male | Wistar rats | *Ex vivo* | Isolated heart perfusion (Langendorff method) | 30 min | 60 min | [32] |
| **Nuñez et al.** | **2014** | Male | Sprague-Dawley rats | *Ex vivo* | Isolated heart perfusion (Langendorff method) | 30 min | 90 min | [44] |
| **Palee et al.** | **2020** | Male | Wistar rats | *In vivo* | Left anterior descending (LAD) coronary artery ligation | 30 min | 120 min | [41] |
| **Pasdois et al.** | **2006** | Male | Sprague-Dawley rats | *Ex vivo* | Isolated heart perfusion (Langendorff method) | 30 min | 20 min | [28] |
| **Ravindran et al.** | **2016** | Male | Wistar rats | *Ex vivo* | Isolated heart perfusion (Langendorff method) | 30 min | 60 min | [29] |
| **Ravindran et al.** | **2017** | Male | Wistar rats | *Ex vivo* | Isolated heart perfusion (Langendorff method) | 30 min | 60 min | [34] |
| **Ravindan & Kurian et al.** | **2019** | Male | Wistar rats | *Ex vivo* | Isolated heart perfusion (Langendorff method) | 30 min | 60 min | [31] |
| **Shi et al.** | **2021** | Male | Wistar rats | *Ex vivo* | Isolated heart perfusion (Langendorff method) | 30 min | 90 min | [42] |
| **Wang et al.** | **2007** | Male | Sprague-Dawley rats | *Ex vivo* | Isolated heart perfusion (Langendorff method) | 20 min | 60 min | [36] |
| **Yang et al.** | **2015** | Male | Sprague-Dawley rats | *In vivo* | Left coronary artery ligation | 30 min | 90 min | [37] |
| **Zuo et al.** | **2009** | Male | Sprague-Dawley rats | *Ex vivo* | Isolated heart perfusion (Langendorff method) | 30 min | 30 min | [38] |

**SI 4 - Drug characteristics**

| **Author** | **Year** | **Primary drug of interest** | **Optimal dose** | **Timing of drug administration** | **Duration of injection/infusion/perfusion time** | **Drug route of administration** | **Ref.** |
| --- | --- | --- | --- | --- | --- | --- | --- |
| **Banu et al.** | **2016** | Sodium hydrogen sulphide (NaHS) | 20 µM | Post-ischaemia | 15 min | Intracoronary perfusion | [30] |
| **Barreto-Torres et al.** | **2012** | Metformin | 2 mM | Before the ischaemic period and during reperfusion | 10 min before ischaemia and throughout the reperfusion period | Intracoronary perfusion | [40] |
| **Bassiouni et al.** | **2023** | ARP-100 and ONO-4817 | 10 µM and 50 µM, respectively | Pre-ischaemia | 10 min before ischaemia and throughout the reperfusion period | Intracoronary perfusion | [39] |
| **Boovarahan & Kurian** | **2021** | 5-Azacytidine | 5 µM | Pre-ischaemia | 10 min | Intracoronary perfusion | [43] |
| **Boovarahan et al.** | **2021** | Sodium thiosulphate (STS) | 1 mM | Both pre-and post-ischaemia | 10 min | Intracoronary perfusion | [31] |
| **Chen et al.** | **2006** | Amobarbital (AMO) | 2.5 mM | Pre-ischaemia | 1 min | Intracoronary perfusion | [27] |
| **Javadov et al.** | **2008** | AVE-4890 | 5 µM | Both pre-and post-ischaemia | 10 min (pre-ischaemia) and 60 min (throughout reperfusion) | Intracoronary perfusion | [35] |
| **Kannan et al.** | **2019** | Sodium thiosulphate (STS) | 1 mM | Both pre-and post-ischaemia | 10 min | Intracoronary perfusion | [32] |
| **Nuñez et al.** | **2014** | Angiotensin II | 10 nM | Pre-ischaemia | 5 min followed by 5 min washout (4 cycles) | Intracoronary perfusion | [44] |
| **Palee et al.** | **2020** | Metformin | 200 mg/kg | Pre-ischaemia | Administered 15 min before I/R protocol | Intravenous administration via femoral vein | [41] |
| **Pasdois et al.** | **2006** | Diazoxide | 100 µM | Pre-ischaemia | 20 min | Intracoronary perfusion | [28] |
| **Ravindran et al.** | **2016** | Sodium hydrogen sulphide (NaHS) | 20 µM | Pre-ischaemia | 15 min | Intracoronary perfusion | [29] |
| **Ravindran et al.** | **2017** | Sodium thiosulphate (STS) | 1 mM | During Reperfusion | 15 min | Intracoronary perfusion | [34] |
| **Ravindran & Kurian** | **2019** | Sodium thiosulphate (STS) | 1 mM | Pre-ischaemia | Administered for 15 min before main ischaemic event | Intracoronary perfusion | [31] |
| **Shi et al.** | **2021** | Sappanone A | 100 µM | During Reperfusion | First 15 min of reperfusion | Intracoronary infusion | [42] |
| **Wang et al.** | **2007** | Cariporide and Metoprolol | 10 µM for both | Pre-ischaemia | 10 min | Intracoronary perfusion | [36] |
| **Yang et al.** | **2015** | 3, 4-dihydroxyl-phenyl lactic acid (DLA) | 5 mg/kg | Before and during reperfusion | 1 min (0.5 ml) before reperfusion and continuously administered until the end of reperfusion (1 ml/h) | Intravenous administration | [37] |
| **Zuo et al.** | **2009** | 5,5-dimethyl-1-pyrroline *N*-oxide (DMPO) | 1 mM | Pre-ischaemia | Unclear | Intracoronary perfusion | [38] |
